# Crosstalk-free optical indicator-actuator combinations for bidirectional optogenetics

**DOI:** 10.1038/s44385-026-00097-3

**Published:** 2026-08-12

**Authors:** Glena Travis, Hallur Reynisson, Anai Gonzalez-Cordero, Stefano Palomba

**Affiliations:** 1 Institute for Photonics and Optical Sciences (IPOS), School of Physics, The University of Sydney, Camperdown, NSW Australia; 2 Stem Cell Medicine Unit, Children’s Medical Research Institute, Sydney, NSW Australia; 3 Neuroscience Research Australia, Sydney, NSW Australia; 4 School of Medical Sciences, Faculty of Medicine and Health, University of Sydney, Camperdown, NSW Australia

**Keywords:** Biological techniques, Engineering, Neuroscience, Optics and photonics

## Abstract

Bidirectional optogenetics enables simultaneous optical readout and manipulation of neural activity. However, optical crosstalk between actuators and indicators impedes clean observation and control. This review surveys genetically encoded indicators and optogenetic actuators, and the mechanisms of crosstalk, then catalogues reported crosstalk-free pairings and trade-offs. Finally, we propose best-practice guidelines and identify future directions, including near-infra-red and two-photon modalities, protein engineering and computational correction.

## Introduction

Optogenetics, a technique enabling the manipulation and recording of neuronal activity with low invasiveness using light-sensitive proteins, has revolutionised neuroscience and biomedical engineering^1–4^. Optogenetics combines light (‘opto’) and genetically encoded proteins (‘genetics’) to drive ion pumps and channels in specific cells, particularly in neurons, without physical electrodes^2–5^. Genetically encoded proteins also enable visualisation of action potentials, referred to as indicators. This is particularly useful in optogenetically manipulated neurons, i.e. in which light-sensitive proteins, actuators, have been inserted, as it enables visualisation of neural activity in response to light. Together, optogenetics and genetically encoded proteins allow precise manipulation and readout of neural activity.

Combining these into all-optical platforms enable simultaneous readout and manipulation, ideally at a single-neuron level with a single-action-potential precision. However, achieving clean bidirectional control is challenging due to optical crosstalk, defined as the unwanted interaction between an optical stimulus (light excitation) and photosensitive actuators outside the intended spatial, spectral or cellular domain, resulting in off-target opsin activation or interference with read-outs showing simultaneous optical signals. Throughout, we treat a combination as crosstalk-free (more precisely, low-crosstalk) when the actuating light produces no statistically significant change in the indicator readout relative to a stimulus-free control at the operating power^6^, equivalently, when any stimulus-locked artefact remains below the indicator’s genuine per-event signal divided by the target signal-to-noise ratio. This threshold is necessarily indicator-dependent: voltage indicators, with small photon-limited single-spike signals, demand a stricter bound than bright, integrating calcium indicators. In this review, we revise in detail the optical crosstalk, examine its mechanisms, catalogue available actuators and indicators, explore engineering strategies to mitigate crosstalk, survey published ‘crosstalk-free’ or ‘minimal crosstalk’ implementations and propose guidelines and open research directions, with a focus on bioengineering innovations for translational applications.

## Engineering principles of bidirectional optogenetics

### Categories of optical crosstalk

**Fig. 1:**
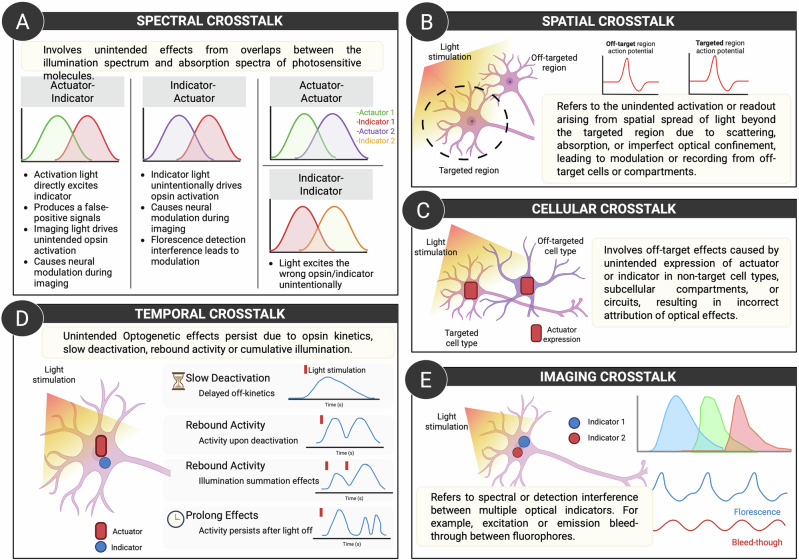
Optical crosstalk mechanisms in optogenetics. **A** Spectral; **B** Spatial; **C** Cellular; **D** Temporal; and **E** Imaging crosstalk. Created in BioRender. Travis, G. (2026) https://BioRender.com/iusxtl2.

Optical crosstalk refers to unintended interactions between optical illumination and photosensitive elements in an optogenetic experiment that leads to off-target neural modulation or contamination of optical readouts. Crosstalk can be divided into five categories, including spectral, spatial, cellular, temporal and imaging crosstalk (Fig. 1).

Spectral crosstalk involves unintended effects arising from overlaps between the illumination spectrum and the absorption or excitation spectra of photosensitive molecules (opsins). It can be divided into further sub-categories^7^. Actuator and indicator crosstalk occurs when light delivered to stimulate an optogenetic actuator (e.g. an excitatory or inhibitory opsin) directly excites or perturbs an optical indicator (e.g. a genetically encoded calcium or voltage indicator), producing artefact fluorescence signals without corresponding neural activity. Similarly, the inverse is also true in indicator-actuator crosstalk, where the light used to excite an optical indicator unintentionally activates an optogenetic actuator, resulting in neural modulation during what is intended to be passive imaging. Actuator-actuator crosstalk results from experiments using two or more optogenetic actuator and the light intended to control one opsin unintentionally activates another opsin expressed in the same or a different neuronal population. This includes interactions between multiple excitatory opsins, or between excitatory and inhibitory opsins, leading to loss of control specificity. Importantly, spectral crosstalk can occur via one-photon or two-photon excitation and is independent of the illumination modality.

Spatial crosstalk refers to the unintended activation or readout arising from the spatial spread of light beyond the targeted region due to scattering, absorption, or imperfect optical confinement, leading to modulation or recording from off-target cells or compartments^8,9^. Cellular/expression crosstalk involves off-target effects caused by unintended expression of actuators or indicators in non-target cell types, subcellular compartments, or circuits, resulting in incorrect attribution of optical effects^10,11^. While temporal crosstalk is when persistence of the optogenetic effects outside the intended stimulation window occurs due to opsin kinetics, slow deactivation, rebound activity, or cumulative effects of repeated illumination^8,12^. And imaging crosstalk refers to spectral or detection interference between multiple optical indicators (e.g. excitation or emission bleed-through between fluorophores). While experimentally relevant, this does not directly modulate neural activity and is therefore typically considered a fluorescence imaging artefact rather than optogenetic optical crosstalk^11,13^.

In conclusion, optical crosstalk in optogenetics encompasses spectral, spatial, temporal and expression-related interactions in which optical illumination unintentionally modulates neural activity or contaminates optical readouts, particularly in experiments combining optogenetic actuators and optical indicators.

### Risks and drawbacks of optical crosstalk

#### Spectral crosstalk

As expected, each crosstalk category leads to specific experimental problems. Spectral crosstalk direct spectral overlap causes unintended activation or inhibition of off-target opsins or excitation of indicators (false stimulation). This confounds which optical input caused the observed neural effect, undermining causal interpretation. Furthermore, in all-optical experiments, stimulator light exciting indicators produces artifactual fluorescence transients (false positives), while imaging light activating opsins produces unintended neural modulation during supposed passive recording.

Hence, spectral crosstalk threatens internal validity since manipulation cannot be dissociated from measurement. Even small overlap at typical illumination powers can produce sizable artefacts, so careful opsin/indicator pairing and wavelength management are essential. Therefore, spectral crosstalk can be mitigated through choosing spectrally orthogonal actuator/indicator pairs, using red-shifted opsins or far-red two-photon windows and empirically measuring cross-activation in controls.

#### Actuator-indicator crosstalk

Actuator and indicator crosstalk can happen when stimulation light excites the indicator or when imaging light activates the opsin (Fig. 1A). The former produces fluorescence transients not caused by neural Ca²⁺/voltage changes; hence, measurement artefacts that inflate apparent activity or produce spurious correlations can be generated. Consequently, it leads to false positives in activity readouts and incorrect inferences about circuit function, compromising the reproducibility of all-optical mapping experiments. When an indicator crosstalks with an actuator, the light used for imaging can drive spiking or suppress activity inadvertently, contaminating baseline measurements and time-locked experiments. Two-photon absorption at typical imaging wavelengths/powers sometimes activates red-shifted opsins or even blue opsins via nonlinear absorption. Consequently, it breaks the key assumption that imaging is non-perturbative; even low-level activation alters network state and biases interpretation of stimulus-response relationships.

#### Actuator-actuator crosstalk

Cross-activation between excitatory and inhibitory opsins leads to actuator-to-actuator crosstalk, which can invert or blunt intended manipulations, produce mixed membrane currents, or require much higher irradiances to achieve reliable directionality. This reduces dynamic range and interpretability (Fig. 1A). Mitigation is fundamental since experiments need bidirectional control or multiplexed population control, because spectral separation and matched photocurrent properties are required to avoid ambiguous outcomes.

#### Spatial crosstalk

The main drawback of spatial crosstalk relates to light scattering and imperfect optics, which can cause off-target activation of nearby cells, neuropil, or axon terminals. Even when the wavelength is well chosen, spatial spread can result in recruitment of unintended populations. Hence, spatial contamination undermines claims about single-cell or projection-specific causality and inflates the apparent effect size of manipulations; critical for cellular-resolution mapping and precise circuit perturbations. To mitigate spatial crosstalk, use two-photon holography or tighter optics, reduce power and map spatial spread empirically (Fig. 1B).

#### Temporal crosstalk

Complications on interpretation of timing-sensitive protocols can come from temporal crosstalk. Opsins with slow off-kinetics, step-function behaviour, or rebound currents generate responses outside intended windows; repeated pulses can summate, producing unintended baseline shifts. Consequently, for causal timing (e.g., millisecond-scale synaptic physiology or behaviourally timed perturbations), temporal bleed invalidates precise attribution of effects to stimuli. It is therefore necessary to choose opsins with appropriate kinetics or design stimulation paradigms to account for kinetics such that interstimulus intervals allow for recovery (Fig. 1D).

#### Expression (cellular) crosstalk

Off-target or ectopic expression (viral tropism, promoter leakiness, retrograde transport) results in expression (cellular) crosstalk, resulting in stimulation of the wrong cells, invalidating cell-type-specific claims. Co-expression of actuator-indicator in the same population can simplify experiments, but also makes crosstalk harder to decompose. Therefore, accurate mapping of function to cell type/projection depends on precise genetic targeting; expression crosstalk is a biological, not optical, source of confound and must be controlled via appropriate viral design (use intersectional genetics or bicistronic constructs when appropriate), controls and histology validation experiments (Fig. 1C).

#### Imaging crosstalk

Imaging crosstalk of indicators due to overlapping excitation/emission spectra produces fluorescence channel bleed-through, misattribution of signals between fluorophores and degraded signal-to-noise (SNR) (Fig. 1E). Unlike optogenetic crosstalk, this does not directly change neural activity but corrupts measurement. Accurate quantitative imaging (e.g. simultaneous calcium + voltage, or green + red GECIs) requires spectral separation, filter optimisation and unmixing; otherwise, population correlations and timing are misestimated.

## Catalogue of engineered actuators and indicators

### Optogenetic actuators (or opsin activators/inhibitors)

Optogenetic actuators are mainly microbial opsins where an ionic current is induced upon illumination. They fall into two categories: excitatory and inhibitory opsins, engineered for spectral shift and sensitivity. Opsins must enable precise spatiotemporal control and high-frequency kinetics^12,14,15^. Table 1 lists spectral and kinetic properties for both excitatory and inhibitory optogenetic actuators.

**Table 1:** Opsin properties

Opsin family	Opsins	Opsin type	Optical excitation/inhibition peak wavelength (nm)	On kinetics (*τ*_on_) milliseconds (ms)	Off kinetics (*τ*_off_)	References
Channel-rhodopsin	ChR2	Excitat-ory	~460	1.7 ± 0.1	10 ± 0.8	^158^
	Chronos		~500	2.3 ± 0.3	3.6 ± 0.2	^18^
	CheRiff		~460	4.5 ± 0.3	16 ± 0.8	^159^
	ChETA		~ 460	4.4 ± 0.4	7.5 ± 0.4	^160,161^
	ChIEF		~460	18 ± 1.8	15 ± 2	^159^
	ST-Chronos		470	N/A	1.7 ± 0.6	^14^
	ST-ChroME		470	N/A	3.0 ± 0.4	^14^
	CatCh		474	0.59 ± 0.003	16 ± 3	^162^
	CoChR		~480	4.5 ± 0.3	86 ± 11	^18^
	ChRmine		~540	N/A	60	^8^
	ChReef		~540	N/A	58	^163^
	C1V1		~539	N/A	156	^164^
	vChR1		~570	~ 2.8	> 90	^165^
	ReaChR		590–630	20.7 ± 0.6	68.1 ± 4.2	^166^
	ChrimsonR		~625	N/A	12.2 ± 0.8	^18,167^
	f-Chrimson		~640	N/A	5.7 ± 0.5	^167^
	vf-Chrimson		~640	N/A	2.7 ± 0.3	^167^
Halo-rhodopsin	eNpHR	Inhibit-ory	~593	6.1 ± 2.1	6.9 ± 2.2	^4,20^
	eNpHR 2.0		~590	7.0 ± 0.5	7.0 ± 0.5	^168^
	eNpHR 3.0		~590	4.2	~5–10	^169^
	Jaws		~590	4.8	7	^170^
Archae-rhodopsin	AR3 (Arch)	Inhibit-ory	~566	N/A	19	^21^
	ArchT		~532	N/A	15 ± 4	^171^
	eArch3.0		520–550	~5– ~8	200	^172^
	eArchT3.0		520–550	2 ± 0.5	~6	^160^
Leptosphaeria rhodopsin	Mac	Inhibit-ory	520–550	2.2 ± 0.5	~40	^160^
	eMac 3.0		520-550	2.0 ± 0.2	~45	^160^
Anion Channel-rhodopsin	GtACR1	Inhibit-ory	~515	N/A	19.1 ± 1.1	^173,174^
	GtACR2		~515	N/A	11 ± 0.8 89 ± 13	^173–175^
	GtACR3		~515	N/A	40 ± 1.9	^174^
	GtACR4		~515	N/A	37 ± 3.0	^174^
	GtACR5		~515	N/A	40 ± 3.4	^174^
Channel-rhodopsin (engineered)	iC1C2	Inhibit-ory	470	N/A	2400	^176^
	SwiChR		470	N/A	7300	^176^
Naturally occurring rhodopsin	NeoR	Inhibit-ory	690	N/A	N/A	^23^

*N/A* Not available.

#### Excitatory opsins

Excitatory opsins are non-selective cation channels that, upon light stimulation, conduct H⁺, Na⁺, K⁺ and Ca²⁺, causing membrane depolarisation and activation^16^. Channelrhodopsin-2 (ChR2), isolated from the green alga Chlamydomonas reinhardtii and the first channelrhodopsin to be widely applied to neurons, transformed the field^1,17^. ChR2 is a light-gated cation channel activated by blue light requiring high intensity, resulting in suboptimal responses compared to its successors^1,17^. Chronos, a blue-light opsin from *Stigeoclonium helveticum*^18^, which offers improved kinetics and light sensitivity with fast on/off-kinetics. Chronos has been used in vision restoration via stable adeno-associated virus (AAV) delivery, providing high temporal resolution and minimising phototoxic risk^19^. Red-shifted opsins like ChrimsonR (625 nm excitation) are paving future human applications^18^ by overcoming blue-light limitations, enabling deeper tissue penetration, reduced scattering and decreased phototoxicity. Notably, as a critical caveat for bidirectional work, spectral red-shifting is not a complete fix for retinal chromophores, as ChrimsonR remains partly activated by shorter-wavelength light, thus reducing but not abolishing spectral crosstalk.

#### Inhibitory opsins

Inhibitory opsins either conduct anions such as chloride (Cl⁻) into the cytoplasm or pump protons (H⁺) out of it; both hyperpolarise the membrane and inhibit cellular activity and inhibition of cellular activity^16^. Halorhodopsin (NpHR) from *Natronomonas pharaonis* translocates Cl^−^ into the cytoplasm under yellow light illumination (589 nm)^20^. Archaerhodopsins (Arch) from *Halorubrum sodomense*, pump H^+^ out of the cytoplasm upon yellow light activation (589 nm)^21^. Jaws, a Cl⁻ pump, is rarely used because a pump translocates only one ion per photocycle, giving a charge transfer per absorbed photon roughly three orders of magnitude below that of light-gated ion channels and unsuitable for long-term inhibition studies due to the constant requirement of illumination, leading to heat damage of cells^22^.

NeoR, a near-infrared naturally occurring rhodopsin (rhodopsin-guanylyl cyclase 3) from *Rhizolosmatium globosum*, has an excitation wavelength of 690 nm and emission at 707 nm^23–25^. Interestingly, when NeoR is illuminated with red light, it converts from its red-absorbing state (NeoR_690_) to a high UV-absorbing state (NeoR_367_), and conversely, UV light illumination converts NeoR_367_ back to NeoR_690_^26^, thereby NeoR exhibits bistable photoswitchable properties. Another study showed that NeoR can mediate an inhibitory response to light when in the presence of rhodopsin-guanylyl cyclase 1/2, resulting in heterodimerisation^23^. Therefore, NeoR has a promising application in optogenetics and in neuroimaging as a near-infra-red opsin and is able to be both excited and inhibited. Furthermore, as the yellow-orange activation of NpHR and Arch overlaps with the excitation of many calcium indicators, NeoR can be highlighted as its near-infra-red operation can be utilised instead for minimised crosstalk.

#### Two photon (2P)/long-wavelength opsins

2P excitation uses longer-wavelength light: near-simultaneous absorption of two photons promotes the fluorophore from the ground state to a higher energy state, allowing for increased imaging depth and reduced phototoxicity compared to single-photon excitation^27^. Although optogenetic actuators, such as ChR, have not been designed by nature to be subjected to 2P excitation, they can still be excited by powerful laser light in the near infra-red spectral region, e.g. with wavelengths spanning from 900 nm to 1100 nm. One study provided a spectral survey of opsins such as ChRmine, ChrimsonR, Chronos, CoChR, ReaChR, ChR2 and ChroME subjected to 2P excitation with wavelengths from 750 nm to 1300 nm. Two-photon action spectra peaked near 1000 nm, with 920 nm illumination sufficient to activate these opsins^8,28^. 2P opsins enable deep-tissue penetration and minimal crosstalk, compatible with advanced microscopy such as computer-generated holography^29,30^, but require powerful lasers.

#### Engineered subcellular-targeted opsins

Localisation of opsins is mainly cell membrane bound^1,17,18,20–22,31^, and different subcellular localisation is also pivotal for reducing off-target effects, improving spatial resolution. Restricting distribution of the opsin into the axonal and dendritic membranes removes the dominant cause of off-target activation due to light scattering or holographic stimulation, which has enabled single-cell-resolution all-optical interrogation at markedly lower power. For example, soma-specific opsins have been developed from the blue-light-sensitive CoChR opsin, soCoChR^28^ and stCoChR^32^, with stCoChR outperforming soCoChR and the original CoChR opsin^32^. Other soma targeting opsins, ST-ChroME and ST-Chronos enable fast kinetics, with St-ChroME requiring less laser power and shorter pulses compared to other opsins^14^. Quantitatively, soma-targeted ChroME was found to deliver 1.8 ± 0.2 nA per unit cell, compared to the 205 ± 50 pA for non-soma-targeted Chronos, which were typically unable to generate action potentials, though soma targeting doubled Chronos elicited photocurrents to 460 ± 60 pA. Thus, further engineering of subcellular targeting opsins further reduces off-targeting effects and thus contributes to reducing crosstalk.

### Genetically encoded indicators

#### Calcium indicators (GECIs)

Genetically encoded calcium ion (Ca^2+^) indicators (GECIs) are widely used in neuroimaging for functional fluorescence signalling. GECIs are based on fluorescent proteins and require external light excitation. When neurons fire, intracellular Ca^2+^ concentration rises, promoting an intramolecular conformational rearrangement in the GECI, changing fluorescence intensity upon irradiation; this can increase or decrease fluorescence as a function of the calcium concentrations, indirectly reflecting neural activity. Scientists use Δ*F*/*F*_0_ to compare calcium traces, where the change in fluorescence (Δ*F*) is divided by the initial fluorescence (*F*_0_). Calcium indicators detect action potentials, but their dynamics depend on ion diffusion and the molecular changes, resulting in slow kinetics. Nevertheless, they exhibit superior fluorescence intensity changes compared to voltage indicators, which can be dim^33^. Table 2 tabulates available GECIs and their properties for experimental design purposes.

**Table 2:** Genetically encoded calcium indicator properties

Type of GECI	Indicators	Excitation peak wavelength (nm)	Emission peak wavelength (nm)	Rise-time (ms)	Half-decay time (ms)	Δ*F*/*F* %, 1 action potential	Kd (nM)	Hill coefficient	Quantum yield (Ca^2+^ bound)	References
GECI Blue	GCaMP8f	496	505–560	6.6	87.5	37	334	2.08	0.64	^34^
	GCaMP6f	496	505–560	22.5	297	20	150	3.10	0.59	^36^
	XCaMP-Gf	492	510	15.6	194	25	154	1.99	0.70	^34,177^
GECI Red	NIR-GECO3.5	656	683	N/A	N/A	-10	298	N/A	0.029	^37^
	NIR-GECO2G	678	704	1200	3000	-16	480	0.78	0.021	^39,178^
	FR-GECO1a	596	642	269	3711	-5.8	29	1.49	0.34	^134^
	FR-GECO1c	596	646	156	6393	-18	83	1.98	0.35	^134^
	RCaMP3	560	610	25.1	145	34	101	2.06	N/A	^179^
	jRGECO1a	560	580	25.5	177	9	102	1.94	0.22	^180^
	XCaMP-R	530-580	600	N/A	~195	20	97	1.1	0.28	^177^

*N/A* not available.

The GCaMP series was the first GECIs used for neural imaging^34^. GCaMP indicators consist of a calcium-binding domain, calmodulin^35^, with one circular permutated GFP molecule^36^. GCaMP6f was among the fastest in 2013, followed by GCaMP8f, which is the current fastest^34^. Red-shifted near-infra-red (NIR) GECIs include the NIR-GECO family: NIR-GECO1, NIR-GECO2 and NIR-GECO2G variants^37–39^. The NIR-GECO family has an absorption peak at ~678 nm and emission peak at ~704 nm^38^, ideal for light penetrating into tissue^39^. NIR-GECO2G is the state-of the art indicator for biocompatibility and Ca^2+^ sensitivity^39^. The main limitations of NIR GECIs compared to other GECIs are low photostability, kinetics and brightness. NIR-GECO3 variants (NIR-GECO3.1-3.5), have absorption at ~640 nm and emission at ~670 nm^37^. NIR-GECO3.5 showed the highest Δ*F/F*0 in cultured neurons and in mice in vivo; however, the NIR-GECO3 series is not substantially improved over NIR-GECO2G in terms of brightness and SNR in vivo.

**Fig. 2:**
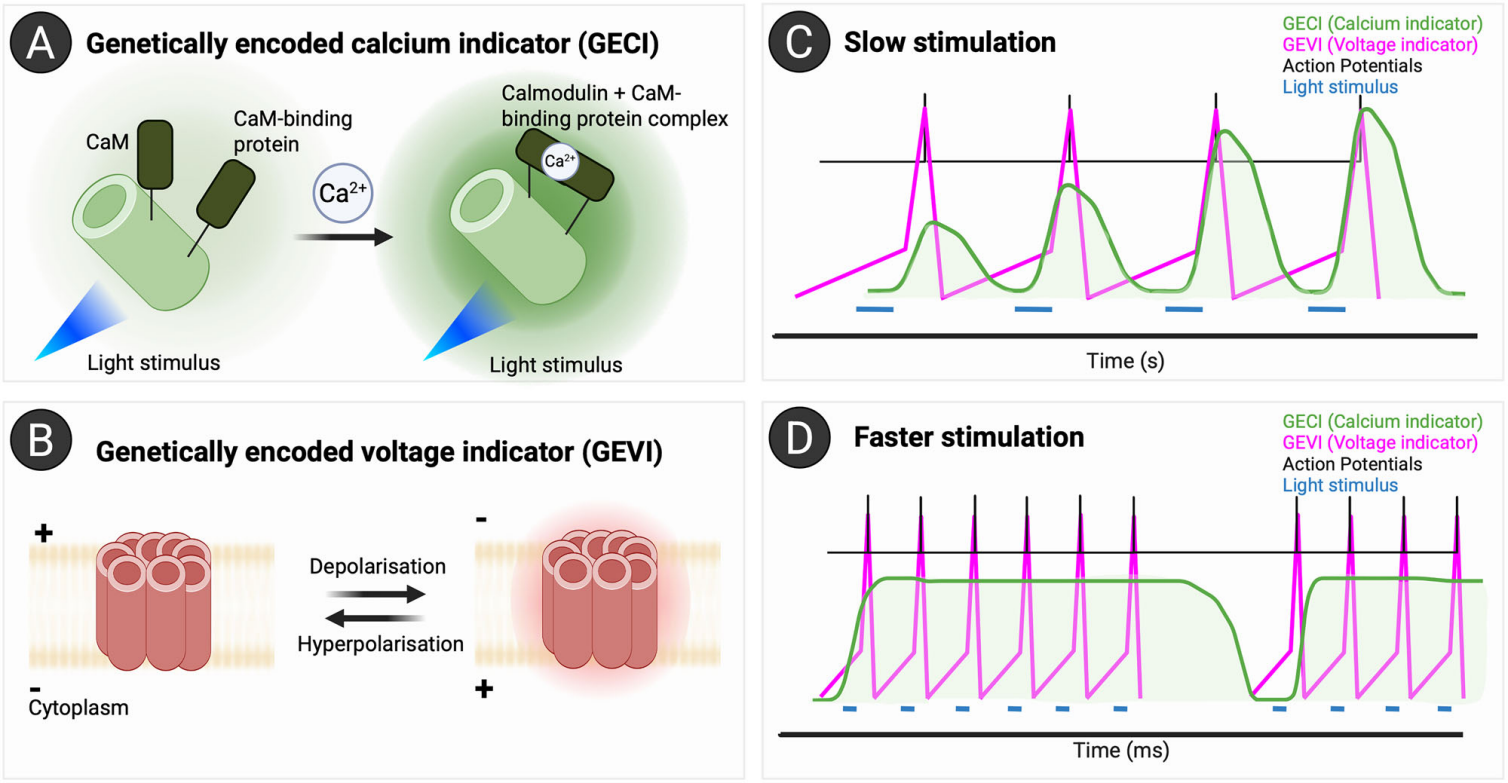
Genetically encoded calcium (GECI) and voltage indicators (GEVI). **A** GECIs work through conformational change in the presence of calcium ions (Ca2+). **B** GEVIs such as Archon1 and Quas families work by detecting membrane potential changes where there are voltage changes. **C** GECI and GEVI readout show a better spiking burst when light stimulation occurs at a slow rate, compared to the calcium trace, which could be missed with light stimulation. **D** Readout at a faster rate light stimulations result in action potentials being lost with GECIs compared to GEVIs. Created in BioRender. Travis, G. (2026) https://BioRender.com/iusxtl2.

Calcium imaging with GECIs captures predominantly action potentials^40^, hence subthreshold excitatory or inhibitory synaptic inputs are invisible. Additionally, biophysical limitations exist as calcium dynamics are significantly slower than membrane potential changes. Detecting individual neural spikes when neurons fire bursts is challenging due to different time scales (1–2 ms versus 100 ms–s). GECIs can change calcium dynamics by altering the intrinsic/extrinsic calcium buffers and affect the interactions between Ca^2+^ diffusion and extrusion^41^. Therefore, calcium imaging with GECIs is limited in capturing the complete picture of neural activity. Voltage imaging overcomes these limitations using GEVIs, enabling accurate decoding of neural activity by precise monitoring of voltage potential changes at the subthreshold level^42^. Furthermore, action potentials detected by calcium indicators require imaging speeds of a few Hertz (Hz) to tens of Hz, compared to voltage indicators needing kHz frame rate for subthreshold changes (Fig. 2).

#### Voltage indicators (GEVIs)

GEVIs offer fast responses to voltage changes^41^ and monitor membrane voltage by sensing the electrical field across the plasma membrane^41^, providing a readout of actual action potentials rather than inferred activity provided by GECI. Different classes of GEVIs exist^43^, including fluorescent microbial opsin-based and GFP-fluorescent proteins coupled to a voltage-sensing membrane protein and electrochromic Forster Resonance Energy Transfer (FRET) eFRET-based designs.

Microbial opsins are dimmer and poorly localised (low SNR) compared to GFP-coupled voltage indicators. Voltage indicators enable complex experiments requiring subthreshold membrane potential monitoring, like synaptic potentials^44^. Opsin-based GEVIs exploit a voltage-dependent fluorescent state of retinal chromophore. Examples include Archs, QuasArs and Archons (Table 3). Some GEVIs are optogenetically incompatible due to spectral overlap, so indicator choice matters.

**Table 3:** Genetically encoded voltage indicator properties

Type of GEVI	Indicator	Excitation peak wavelength (nm)	Emission peak wavelength (nm)	Depolarisation −70 to 30 mV τ-fast (ms) at 32°C-37°C	Repolarisation 30 to −70 mV τ-fast (ms) at 32°C-37°C	Δ*F*/*F* %, 1 action potential	Quantum yield (Ca^2+^ bound)	Reference
GEVI VSD-based	ASAP5	440–480	530	0.8	1.1	−40	N/A	^181^
	ASAP3	440–480	530	1.3	3.5	−20	N/A	^181^
	ASAP4e	480	503	1.5	3.66	−38	N/A	^182^
	JEDI-1P	484	509	0.54	1.2	−30	N/A	^183^
	JEDI-2P	940	1100	0.54	1.2	−49	N/A	^91^
	SpikeyGi2	920	N/A	N/A	29.3	58	N/A	^184^
GEVI Microbial rhodopsin-based	Arch	558	687	< 0.5	1.9	40	0.0009	^185^
	Archon1	637	640	0.61	1.1	60	N/A	^44^
				2.2	1.6	70	N/A	^186^
GEVI eFRET-based	QuasAr6a	635	710	1.8	1.3	73	N/A	^186^
	Ace2-mNeo	509	544	0.37	0.50	−18	N/A	^48^
	Ace2-mNeo2	509	544	0.77	0.81	−26	N/A	^49^
	VARNAM	488	580-600	0.88	0.80	−12	N/A	^187^
	VARNAM2	455	480	0.50	0.48	−19	N/A	^49^
	Voltron _525_	530	625	0.85	4.8	−10	N/A	^51^
	Voltron2 _525_	530	625	0.67	3.3	−6	N/A	^51^
	Positron	510	660	0.63	0.64	18	N/A	^188^
	pAce	650	660	0.51	0.61	31	N/A	^49^
	pAceR	650	660	2.4	1.9	28	N/A	^49^
	nirButterfly	640/670	702/720	0.58	17.5	~34	N/A	^189^

*N/A* not available.

eFRET-based GEVIs are another class where fluorescent proteins and other chemical fluorophores, due to microbial rhodopsin absorption spectrum overlaps with the emission spectrum of fluorescent proteins and can serve as FRET donors, while rhodopsin molecules can serve as FRET acceptor^45^. Changes in absorption of rhodopsin are detected by eFRET sensors through quenching of an attached fluorescent protein’s intensity. FRET-opsin-based GEVIs detect voltage depolarisation by decreasing the emission intensity from the fluorescence donor^46,47^. Opsins such as Mac and Ace2 were successfully used to generate indicators with faster kinetics and high dynamic range^46,48,49^. Synthetic fluorescence dyes as eFRET donors are also available to be used with opsins such as Voltron with a HaloTag domain present^50,51^. Advantages of these synthetic dyes during in vivo 1P voltage imaging are that they are more photostable and brighter than fluorescent proteins^47,51–53^. However, both rhodopsin- and eFRET-based GEVIs tend to reduce voltage sensitivity under 2P excitation^54^.

Archon1, a red-light voltage reporter, is derived from a light-derived proton H^+^ pump. It has been shown to have excellent membrane localisation in cultured mouse neurons^44^. Piatkevich and colleagues^44^ used Archon1 to image mouse cortical brain slices for synaptic responses and tested spiking and subthreshold activity in zebrafish; and optogenetically manipulated *C.elegans* neurons, showing markedly improved brightness and membrane localisation, and enabled high-fidelity imaging of action potentials and subthreshold voltage dynamics.

HEK293FTcells expressing Archon1 with >100-mV swings showed 80% Δ*F*/*F* versus template at 40% and Archon2 at 20%^44^. Photobleaching is another factor to consider when utilising voltage indicators, Archons excited for 900 s retained 95% baseline fluorescence. FRET-based nirButterfly provides higher SNR than GFP-coupled voltage sensors^55^. nirButterfly uses a donor 640/670 nm and acceptor 702/720 nm^55^.

Advances in GEVI engineering allow specific targeting and measurement of neuronal activity in distinct types or subcellular compartments. Localisation of GEVI is pivotal for accurate recording of the membrane potential change. soma-monArch restricts the indicator to the soma, achieving a ninefold increase in brightness relative to monArch, improving precision^56^. Archon3 achieves 5-fold brightness compared to Archon1^56^. Archon1 exhibits excellent membrane localisation with improved SNR^44^, but not soma localisation. Despite advancements in calcium and voltage imaging via GECIs and GEVIs, patch-clamping remains the gold standard for measuring ionic current and membrane potential of a targeted neuron at a time^57,58^.

### Delivery of actuators and indicators

#### Overview and selection considerations

The delivery of the optogenetic actuator and indicators is pivotal for successful neuroscience experiments and future clinical applications. Opsin genes expression occurs via three primary methods: viral transduction, transgenic or knock-in-mouse lines or Clustered Regularly Interspaced Short Palindromic Repeats (CRISPR) knock-in human induced pluripotent stem cell (iPSC) lines and Cre recombination^59^.

#### Viral transduction

Traditionally, viral vectors efficiently deliver genetic material into nuclei with minimal adverse effects^60^. Common forms include AAVs, lentiviruses (LVs) and herpes viruses with specific or non-specific promoters^60^. AAVs are preferred for optogenetics due to stability, low immunogenicity and ability to deliver small sequences (<4.7 Kilobases) into post-mitotic cells (cardiomyocytes or neurons) without host genome integration^60^. AAV serotypes differ in cell-type specificity, transduction levels and spatial spread. For example, the dorsal root ganglion consists of a heterogeneous sensory-nerve population, comprising afferents of varying modalities and diameters, leading to challenges for optogenetic application, AAV9 successfully expressed ChR2 in non-transgenic rats dorsal root ganglion^61^. Recombinant AAVs (rAAVs) have been engineered to address neuronal damage, immunogenicity and low transduction efficiency^62,63^. LVs are mostly used to generate cell lines as they integrate into the host genome and are suitable for mitotic and stem cells with larger packaging capacity (8–10 Kilobases), but transduction is slower in terms of speed/diffusion^64^. Opsins and/or indicators are delivered via viral transduction or plasmid/s transfection. Neuronal expression depends on the method, but robust AAV expression typically takes 2–3 weeks^65^.

#### Non-viral transduction

Non-viral methods include physical delivery by electroporation. Bulk electroporation is fast and works in most cell types but is not cell-type selective and reduces viability; single-cell electroporation^66,67^ is precisely targeted but low in throughput, and in vivo gene delivery has safety concerns attributed to its use^68^. Chemical delivery of non-viral optogenetics via lipid-based transfection, which is suitable for most cell types; however, transfection is non-specific and results in low transfection efficiency compared to viral transduction^69^. Another method is the tandem-cell unit system, in which non-excitable opsin-expressing (donor) cells are co-cultured or delivered with host cells, such as cardiomyocytes, where the two cells become electrically connected via gap junction coupling so light-induced donor activity triggers host-cell responses, making the tissue optically excitable^70^. This method enhances safety compared to viral methods and is energy-efficient; however, it relies on successful cell coupling, which can be affected by pathology, and introducing donor cells present in vivo challenges^70^. Nanoparticle-mediated delivery using carbon or gold platforms is complex but allows ligand-mediated cell-specific binding, providing high selectivity, fast expression and sustained opsin release with potential for in vivo use^71,72^.

However, delivery of larger opsin and indicators is problematic; knock-in-mouse lines and Cre recombination^59^ generating transgenic lines^73–75^ mitigate this. These constructs achieve specific, stable opsin/indicator expression^76^. Precise genome engineering is achieved via the Cre-loxP recombination system, however, the existing photoactivable Cre tools lack DNA recombination efficiency, background activation, slow activation kinetics and poor tissue penetration^77^. The Cre-loxP system works via a site-specific Cre recombinase, an enzyme that recognises and binds to specific DNA sequences called loxP sites. DNA between two loxP sites can be deleted or inverted depending on the orientation of the loxP sites, therefore genes can be turned off or modified using this conditional gene editing tool. Red-light controlling Cre systems are rare as they require continuous illumination to achieve DNA recombination^78,79^. REDMAP_Cre_ is the latest red-light-controlled split-Cre system that enables rapid activation at 1-s illumination with an increase in recombination efficiency^77^. Transgenic mouse lines were generated and validated in multiple organs using REDMAP_Cre_^77^. Site-specific transgenesis in other mammalian species is difficult and costly^80,81^. Therefore, transgenic lines for experimentation might not be practical, hence the need for non-germline modified enhancements in AAVs and other viral or non-viral technologies^82^. Additionally, the generation, validation, and maintenance of transgenic lines is expensive and laborious.

Recently, iPSC lines have been engineered using CRISPR^83,84^ to constitutively express opsins. One study inserted f-Chrimson, a red-light activatable channel under the control of the Synapsin promoter, into the safe harbour AAVS1 locus via CRISPR/Cas9 and used multielectrode arrays (MEAs) on cardiomyocytes for validation^83^. Another study utilised the same strategy using Jaws, a red-light-sensitive chloride pump^84^. In this case, Jaws was controlled by the Crx promoter, driving specificity to photoreceptor cells^84^. This offers a promising therapeutic approach aimed at transplanting photoreceptors that are artificially sensitive to light due to the expression of Jaws for vision restoration in photoreceptor degenerative diseases^84^. More iPSC optogenetic lines are expected to be engineered as new tools useful for future disease model studies. Table 4 provides a comparison of viral transduction, CRISPR knock-in and non-viral transduction.

**Table 4:** Side-by-side comparison of delivery techniques for opsin and indicator expression

Feature	Viral transduction		CRISPR knock-in		Non-viral transduction	
Expression longevity	✓	Not lost in post-mitotic cells such as neurons^190,191^	✓	Permanent due to genomic integration^76,192,193^	!	Persists in post-mitotic neurons but episomal cargo declines over weeks; transient in dividing cells^66,67^
Differentiation survival	✓	Not lost in post-mitotic cells such as neurons^84^	✓	Genomic knock-in is inherited through cell decision and differentiation^193^	✗	Episomal cargo lost through division unless an integrating system (transposon/integrase) is used
Expression uniformity	✗	Mosaic expression with variable per-cell transduction efficiency^190^	✓	Homogeneous expression by selecting homozygous knock-in clones^193^	!	Variable with transfection efficiency, but co-delivered transgenes share one payload: consistent actuator:indicator ratio per transfected cell
Genomic safety	!	Random integration may cause insertional mutagenesis^60,191^	✓	Targeted, reducing off-target genotoxicity^193^	✓	Largely episomal: low insertional-mutagenesis risk; nanoparticle biocompatibility must be assessed
Speed	✓	Fast production and expression (days to a week)^191^	✗	Slower, requiring Homology-directed repair-mediated integration, clone selection, validation over weeks^193^	✓	Fast (hours-days); no viral production needed
Flexibility	✓	Higher: delivery easily across cell types^191^	✗	Lower: each locus and insert requires specific donor template design^80,81^	!	Lower intrinsic tropism; relies on promoters, electroporation site, or ligand-functionalised carriers
Clinical relevance	!	Limited: concerns around immunogenicity and random integration^190,191^	✓	Higher: precise edits reduce off-target risks, several clinical homology-directed repair trials underway^190^	!	No comparable cargo ceiling; large or multiple transgenes co-delivered in one payload
Cargo/packaging capacity	!	AAV ~ 4.7 kb ceiling: actuator + indicator + promoter often exceeds ceiling, forcing dual-vector co-injection; lentivirus ~8–10 kb^191^	!	Insert size limited by HDR efficiency; large knock-ins inefficient	✓	No comparable cargo ceiling; large or multiple transgenes co-delivered in one payload^66^
Relevance to crosstalk-free design	!	Dual-AAV co-injection sets an uncontrolled opsin-indicator ratio; variable cellular (expression) crosstalk	✓	Single-copy, defined-locus expression gives the most uniform per-cell stoichiometry (lowest expression crosstalk), at the cost of speed/multiplexing	✓	Co-delivery of both transgenes on one construct at a defined ratio gives the tightest control of the expression ratio that governs cellular crosstalk; any nanoparticle/upconverting carrier must also emit clear of the indicator readout

## Technological advances in optogenetic systems

Multiple imaging techniques have emerged in the field of optogenetics in the last decade, but live tissue imaging remains challenging due to heterogeneity, causing light scattering, absorption and depth issues. Single-photon (1P) microscopy with digital mirrors was previously used for single-target and multi-target excitation in head-mounted animal experiments and in situ applications, but only superficial brain layers (~250 μm) could be imaged; deep-tissue imaging was not possible^85^. Multiphoton fluorescence microscopy using 2P^86^ and 3P^87^ established the next frontier in guiding light through tissue and deep-tissue imaging of large neural populations in behaving animals with high-spatial temporal resolution, even at single-cell or spine resolutions^85^.

### 1P, 2P and 3P fluorescence imaging

**Fig. 3:**
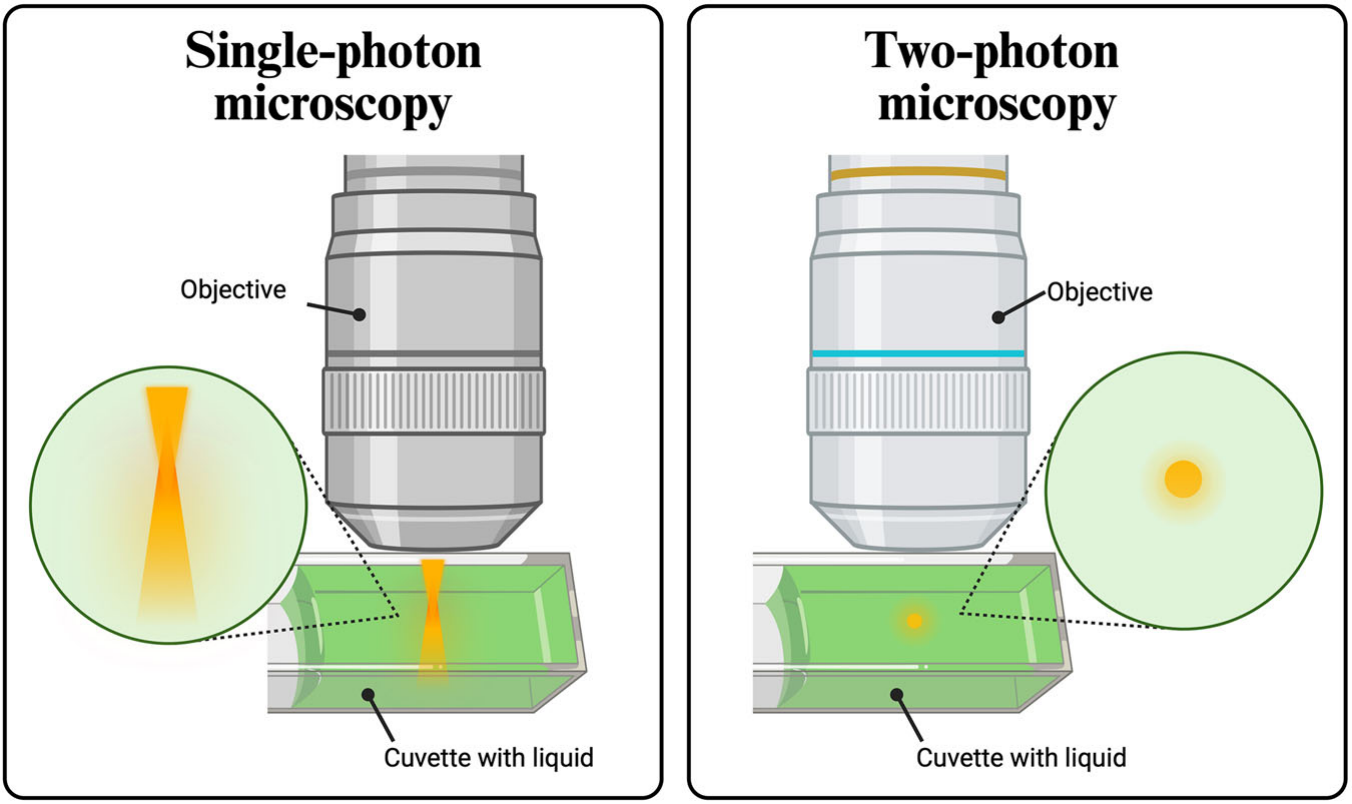
Single-photon and two-photon microscopy comparison with interaction with liquid medium. Single photon emission occurs through a large volume potentially exciting everything in the cone of light, including background. Two-photon emission is concentrated to a much smaller volume, leading to lower background. Created in BioRender. Travis, G. (2026) https://BioRender.com/iusxtl2.

2P imaging uses intense longer wavelengths, achieving 2–3 times the tissue penetration of 1P confocal microscopy; 3P allows even greater penetration with longer wavelength^27^. 2P imaging works when fluorophores absorb two photons simultaneously and emit light on return to the ground state. For instance, an excitation of 450 nm (1P) corresponds to ~900 nm (2P) and ~1350 nm (3P), when three photons interact simultaneously. 1P imaging (widefield or confocal) generates a high background since all light in the objective cone excites molecules (Fig. 3). 2P mitigates this because two photons coincide only at the focal plane, reducing background^27^. Out-of-focus fluorescence is rejected without a pinhole, unlike 1P confocal microscopy^27^. 3P uses three photons at ~1300–1700 nm for deep brain imaging versus <1060 nm for 2P imaging. Spatial resolution is slightly worse in 3P, but the out-of-focus background is far lower compared to 2P due to longer wavelengths^27^. Furthermore, 3P microscopy has been utilised to image subcortical structures in vivo through the intact skull^87^. Despite this, limited fluorophores for 3P restrict depth imaging^27,88,89^.

### Imaging for optogenetics

The gold standard for imaging neurons and neuronal activity is 2P-Laser-Scanning Microscopy, where a photodetector measures fluorescence generated by scanning the excitation focus across a sample^27^. Whilst for an all-optical 2P experiment, a near-infra-red NIR pulse laser is used for optogenetic photostimulation. For deep tissue imaging, 2P delivers ~25 nJ pulses at ~80 MHz repetition rate and wavelengths <1100 nm^27^; 3P requires higher power and can induce tissue damage^27^. To avoid thermal nonlinearities, 3P uses 1 μJ pulses at ~1 MHz within 1300 nm–1700 nm^27^ reducing scattering and absorption within brain and other organic tissues, enhancing SNR by minimising out-of-focus fluorescence. 3 P penetration reaches 1–1.3 mm in brain tissue compared to 0.6–0.8 mm using 2P, however, 3P is expensive, making it only useful when 2P microscopy fails^27^.

In optogenetics, utilising voltage indicators in highly light-scattering tissues, such as brains, remains challenging. 1P microscopy with far-red excitation voltage indicators reduces background from scattering light^90^. 2P transformed calcium imaging, and recent works apply 2P to voltage indicators^91,92^. Despite brighter FRET-opsin GEVIs, detecting voltage-induced fluorescence changes under typical 2P illumination has been challenging^93,94^. A recent study using FRET-opsin GEVIs, Voltron1 and Voltron2, thanks to an optimised protocol by using effective 2P excitation of the dye while undergoing efficient FRET with the opsin, demonstrated successful high-speed 2P voltage imaging in vivo in the mouse barrel cortex^53^.

2P imaging differs from 2P stimulation, where opsins rely on slower ion exchange via opening and closing of ion channels based on kinetics, compared to fluorophores. The ion channel needs to open once to function as an opsin, however, the typical 2P fluorescence imaging provides 80 million pulses per second. Therefore, laser illumination with a lower repetition rate is needed for optogenetics stimulation^95^. Additionally, the soma to be addressed (typically ~10 μm across) is much larger than the diffraction-limited 2P focal spot, so the excitation must be scanned over, or spatially enlarged to cover, the cell. Advancements in 2P optogenetics stimulations have been expanding.

Selective photostimulation of neurons of interest has been achieved by using 2P stimulation in combination with light shaping techniques, where the laser beam is split into multiple spots, simultaneously illuminating targeted neurons. These techniques, such as Computer-Generated Holography (CGH)-2P-CGH-enables faster responses to optogenetic stimulation. However, higher peak power is needed and neurons located above or below the focal plane are not addressable, which is a limitation of the technique. Hence, the field has moved to three-dimensional (3D) scanless holographic optogenetics with temporal focusing (3D-SHOT). 3D-SHOT is a photostimulation technique that relies on temporal focusing: a diffraction grating disperses the spectral components of the pulse so that it is recompressed in time, and therefore reaches peak intensity, only at the focal plane, suppressing out-of-focus two-photon absorption. The most recent advancement has combined 3D-SHOT with large FOV 2P microscopy for optogenetics stimulation and imaging enables the control of more neural activities^96^.

## Wired and wireless LED optogenetic devices

Efficient light delivery is essential for optogenetics stimulation. Two main light sources are used: coherent light (lasers) and incoherent light (micro-sized light-emitting diode (µLED))^59^. Lasers provide high-intensity, minimal beam divergence and precise targeting of deep neural structure with low scattering^59,97^. However, laser systems are tethered to optical fibres, limiting portability in free moving animals^98^. They also require high power and external components for long-term implants^99^.

The field now favours wireless, low-power, compact devices using µLEDs^100–103^. µLED light delivery occurs via waveguides or direct integration. Waveguides offer controlled propagation and precise spatial targeting with minimal off-target illumination, but couple light less efficiently than direct integration^104,105^. Waveguides can integrate with MEAs for simultaneous optical stimulation and neural recording^106–108^. Being passive, waveguides avoid local heating, unlike directly integrated µLEDs.

Optical-electrode (Optrode) devices combine light sources (fibres or LEDs) with electrophysiological recording, enabling simultaneous neuronal activity monitoring. They allow precise modulation of deep-brain circuits while minimising tissue damage^109^. A sapphire-based optrode provided low-noise recordings and efficient light coupling^110^. In optogenetics applications, optrodes facilitate closed-loop systems for studying behaviours in free-moving rodents or non-human primates, with designs like the Utah Optrode Array incorporating µLED for enhanced illumination and wireless integration to support chronic implants^111^. Coaxial and fibre-tipped variants further refine control over light propagation and electrode proximity, reducing artefacts and improving signal fidelity in multifunctional probes for advanced neural interfacing^112^.

The field is moving towards optogenetic neural probe devices; a recently designed device delivered light via µLED and fluids via a microfluidics channel in a single device implanted into mice that minimises invasion, delivers the opsin with precision and does not cause tissue damage upon illumination^113^. The optogenetic neural probe device surpassed the conventional optrode devices during motor activity stimulation tests performed across several weeks, where significantly more movement and higher distance travelled long-term were observed when stimulation was performed using the probe device compared to optrode device^113^. These advances in the delivery of both light and fluids are at the frontier of what can be achieved for future optogenetic applications.

### Upconversion nanoparticles for optogenetics

Visible light penetration into tissue has limitations, e.g. low tissue penetration, high absorption and scattering. Indeed, light scattering in tissue is dominated by Mie scattering (from cells, organelles, collagen fibres, etc.), with a wavelength dependence approximately:

$${\mu }_{s}^{{\prime} }\propto {\lambda }^{-b}$$

where ${\mu }_{s}^{{\prime} }$ is the reduced scattering coefficient, λ is the wavelength (usually in nm or µm) and b≈0.7−1.5 is the scattering power exponent, which quantifies how fast scattering decreases with the wavelength; it is an empirical exponent that captures how tissue microstructure causes scattering to fall with wavelength and it is a tissue- and structure-dependent parameter. So, as wavelength increases, scattering decreases. Hence, shorter wavelengths generate stronger scattering and shallower penetration^27^.

One way to mitigate this issue is to implement two-photon (2P) excitation (~900–1100 nm), which exploits low scattering, higher tissue penetration and confined excitation volume; this is the reason why 2P excitation is considered the gold standard for deep, precise in vivo work^27^.

Another alternative is offered by up-conversion nanoparticles (UCNPs), which convert NIR wavelengths locally into visible light^114–118^, even if the process exhibits very low quantum yield. Indeed, UCNPs absorb NIR light that penetrates tissues much better than visible light because scattering is lower at NIR wavelengths and absorption by water/blood is moderate in the so-called ‘optical/NIR window’, i.e. 650–1350 nm. UCNPs then up-convert this energy into visible emission (e.g., blue/green) near the opsin’s activation band, i.e. enabling opsin stimulation deep below the surface^119^.

Nevertheless, there are some limitations as well with UCNPs. Their quantum yields under continuous illumination are low (0.1–9%)^120^ and require high NIR laser fluency, which could lead to artifactual results due to brain heating leading to altered neuronal activity^121^. However, with optimised host/dopants and sensitisers, they can still elicit biological responses^116^. Moreover, efficiency and heating effects remain practical issues at higher powers. Thus, UCNPs are a viable optical deep stimulation strategy, complementary to 2P excitation, which uses nonlinear absorption instead of up-conversion. One study utilised these specialised UCNPs injected into mice brain that upconvert near-infra-red light from outside of the brain into blue light that activates the ChR in dopaminergic neurons at a depth of ~ 4 mm^119^.

### Photoacoustic methods

Photoacoustic effects can be used for both neuromodulation and imaging. For neuromodulation, pulsed light is used to cause neural activation through mechanical or thermal effects, at higher precision than piezo-based low-frequency ultrasound and at a much lower energy cost than direct photothermal stimulation^122^. One study used nanosecond laser pulses via photoacoustic nanotransducers based on semiconducting polymer nanoparticles to induce acoustic waves via light stimulation, and upon injection into the brain cortex of the freely behaving mice to modulate brain activity^123^. Although these light pulses are short bursts, and with energy ranging from a few μJ to tens of μJ, the light-absorbing transducer itself must still be kept spectrally clear of present genetically induced indicators or actuators. Alternatively, spectroscopic Functional Optoacoustic Neuro-Tomography (sFONT) is a non-invasive deep tissue neural activity imaging technique using both light and ultrasound^82,124^. FONT achieves millisecond temporal resolution and sub-millimetre spatial resolution without harmful temperature accumulation. Another study characterised the calcium indicator NIR-GECO2G in vivo using functional widefield imaging sustained for over several days with high SNR^125^. FONT was used to demonstrate NIR GECIs achieving >5 mm imaging depth^125^. A recent study successfully imaged deep-tissue (~7 mm) with high sensitivity using a biliverdin reductase knockout in a diabetes mouse model^126^.

### Magnetic-field-mediated light sources for optogenetics

Magnetoelectric transducers are wireless electrical neuromodulation techniques, using nanoparticles of multiferroic material to convert magnetic fields into electric fields. Their use for stimulating deep brain targets in Parkinson’s disease has so far been explored computationally^127^. Another study combined the magnetostrictive properties of cobalt ferrite with the ferroelectric properties of barium titanate in composite nanoparticles driven by magnetic fields to modulate neural activity in deep brain regions^128^, through biocompatibility remains a concern. A recent study developed biocompatible polyvinylidene fluoride (PVDF) nanofibers embedded with magnetite nanodiscs, enabling wireless magnetoelectric neuromodulation in ex vivo human brain tissue and in free-moving mouse brains^129^. However, replication with larger sample numbers and long-term stability testing is needed; despite this, magnetoelectrical nanofiber implants offer advantages as flexible, less invasive alternatives to conventional rigid implantable electronics^129^. Conceptually, magnetoelectric and other field-driven approaches address crosstalk by sidestepping it: because actuation is triggered by magnetic rather than optical energy, it does not share a channel with optical indicators and so cannot produce optical actuator-indicator crosstalk, although slow kinetics currently limit the temporal precision achievable in all-optical-style experiments.

## Development of genetically encoded indicators

The development of genetically encoded indicators with enhanced brightness and sensitivity relies on protein engineering and directed evolution. Recent GEVI variants, such as Archon3 and monArch, enable monitoring neural activity with higher brightness^56^. Currently, opsins and indicators are engineered using directed molecular evolution and screening libraries via automated microscopy^56^. The 3D structure of new opsins and indicators are assessed^56^ using predictors like AlphaFold3^130^, as done with Arch-derived GEVIs to identify changes improving Archon3 and monArch performance^56^.

The GCaMP-series of GECIs were designed to link calmodulin (CaM) and target peptide (M13) with a fluorescent protein at the end^36^. A different strategy added CaM and M13 between the fluorescence protein, mNeonGreen^131^, creating NCaMP7^132^. Further development using mNeonGreen produced NEMO^133^, a GECI with higher brightness and larger dynamic range than NCaMP7. As far-red calcium indicators are needed due to deep-tissue penetration, reduced photobleaching and simultaneous use with optogenetic tools, FR-GECO1a and FR-GECO1c were developed using far-red proteins mKelly1 and mKelly2^134^. Therefore, protein engineering combined with novel fluorescent proteins and directed evolution will drive further advancements in actuators and indicators.

## Strategies for crosstalk-free combinations

### Selection of opsin and indicator

**Fig. 4:**
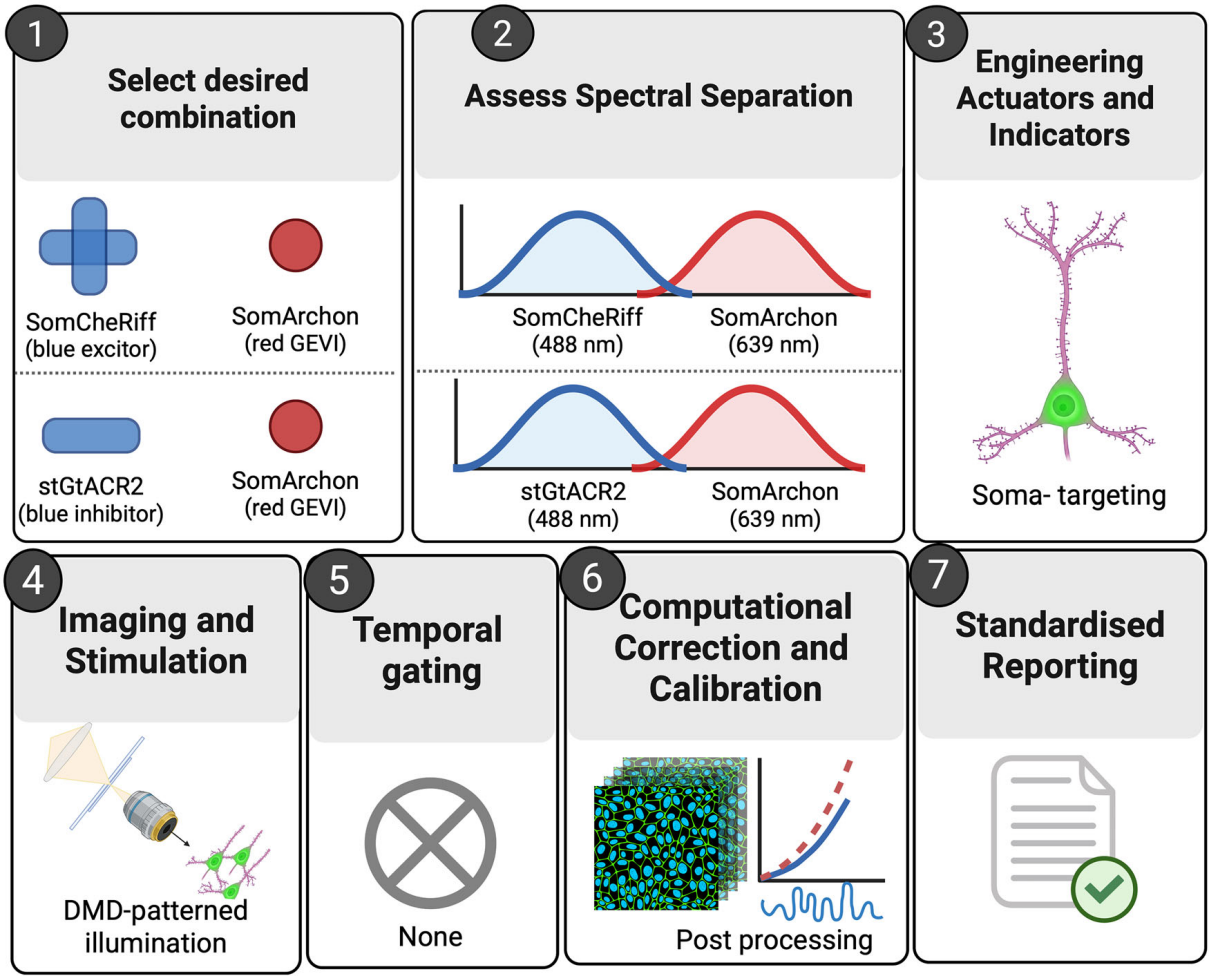
Strategies for crosstalk-free bidirectional optogenetics, illustrated by a worked example. Each numbered panel is a step in selecting and operating a crosstalk-free system, here instantiated by an all-optical platform that excites, inhibits and voltage-images the same layer-1 cortical neurons in vivo without detectable cross-activation reported by Fan et al.^151^ **(1)** Selecting the desired combination: a bidirectional design pairs two blue-light-activated, soma-targeted actuators: the excitatory channelrhodopsin SomCheRiff (blue plus sign) and the inhibitory anion channelrhodopsin stGtACR2 (blue minus sign) with a single red-excited, microbial-rhodopsin-based genetically encoded voltage indicator (GEVI), SomArchon (red circle). **(2)** Assess spectral separation: Both actuators were highly spectrally separated from the indicator as could be excited at 488 nm and 639 nm, respectively. **(3)** Engineering: restricting expression of both actuators and the indicator to the somatic membrane limits neuropil and out-of-focus contamination, to which thin, membrane-delimited voltage signals are especially vulnerable (cellular crosstalk). **(4)** Imaging and stimulation: holographic structured illumination with a digital-micromirror device (DMD) patterns the 488 nm stimulation onto selected somata, while confocal-like excitation and spatially filtered emission rejects out-of-focus signal (spatial crosstalk). **(5)** Temporal gating: not employed by Fan et al., who used continuous kilohertz voltage imaging leaving little interleaving headroom. **(6)** Computational correction and calibration: digital spatial filters were applied in post-processing to separate each cell’s signal from background, and the operating window was calibrated empirically. **(7)** Standardised reporting: wavelengths, irradiances and targeting strategies were reported in full to permit replication. Created in BioRender. Travis, G. (2026) https://BioRender.com/iusxtl2.

The foundational step to minimising optical crosstalk is through thoughtful selection of actuators or opsins and fluorescence indicators with minimal spectral overlap (see Figs. 4-1 and 4-2). Actuators vary in action spectra, kinetics and light sensitivity, while indicators differ in excitation/emission profiles and photostability. Pairing an actuator and reporter with maximally separated excitation bands reduced inadvertent activation or bleaching during imaging. Additional consideration includes brightness, SNR and kinetic compatibility for fast neuronal circuits where reporter kinetics must match the timescale of actuation. Quantitative values for these parameters, such as excitation and emission maxima, activation and decay kinetics, fractional fluorescence change per single spike, Kd and quantum yield, are collated in Tables 1–3. Crosstalk can substantially be reduced by combining blue-sensitive opsins such as ChR2, Chronos, with red or far-red indicators such as jRGECO1a or RCaMP variants. Conversely, long-wavelength-activating opsins such as ChrimsonR and red-shifted ChRmine variants can be paired with green indicators, provided the opsin’s short-wavelength absorption tail does not overlap the imaging wavelength. Soma-targeting or neurite-restricted opsins further decrease inadvertent activation of neighbouring compartments or axons (see Figs. 4-3). Spectral unmixing approaches can be reached by acquiring the full lambda stack and applying linear unmixing or non-negative matrix factorisation to mathematically suppress residual crosstalk when spectral separation alone is insufficient.

More formally, spectral unmixing assumes a linear mixing model in which the signal measured across detection channels equals the sum of each fluorophore’s reference emission spectrum weighted by its abundance (m = Sc, where S is the matrix of reference spectra). Linear unmixing recovers the abundances by least-squares inversion of S, which is well-posed only when the number of detection channels is at least the number of spectrally overlapping fluorophores and S is well-conditioned; as spectral overlap increases, S approaches singularity and the inversion amplifies shot noise, so unmixing trades crosstalk for reduced signal-to-noise rather than removing it outright^135^. Non-negative matrix factorisation relaxes the need for known reference spectra by estimating the spectra and abundances jointly under a non-negativity constraint, but its factorisation is not unique unless additional sparseness or spatial-footprint priors are imposed, the reason constrained-NMF variants are now standard for demixing spatially overlapping sources in functional imaging^136^.

The choice between calcium and voltage indicators directly shapes the crosstalk budget of a bidirectional experiment. GECIs integrate calcium transients over tens to hundreds of milliseconds, leading to single-spike fluorescence which decays with a half-time of roughly 140 ms for fast calcium indicators, like GCaMP6f, and 550 ms for slower ones, like GCaMP6s^36^, and as such they are usually recorded at around 20–30 frames per second with low duty-cycle illumination, due to their high brightness, and even the fastest current indicators, such as jGCaMP8f, with a half-rise of around 7 ms, remain limited by the intrinsic slow nature of calcium decay^34^. Such temporal smearing, coupled with the high signal-to-noise ratio give a comparatively wide margin in which brief stimulation artefacts can be temporally gated or averaged out, although the same integration prevents resolution of single spikes and subthreshold events, though computational methods attempt to de-convolve calcium traces to their underlying spiking sequences^136–139^. GEVIs, by contrast, report sub-millisecond voltage transients, with onset and decay time constants of around 1 ms for chemigenetic indicators such as Voltron^52^, leading to higher sampling rates around 0.5–1 kHz with continuous, often high-intensity illumination, which leaves little temporal gap for interleaving, and undesirable faster bleaching of the indicator, compounding their smaller photon budget. Furthermore, their single-spike responses are also markedly smaller and are photon-limited, with ASAP3 showing optical spikes with an average peak amplitude of 8.8%^140^. However, voltage indicators grant the experimenter with the benefit of monitoring sub-threshold activity, much like patch clamping. Furthermore, since GEVIs are reporters of localised voltage activity, they are more prone to spatial crosstalk due to micro-motion displacement of imaging voxels, whereas soma-filling calcium signals are comparatively motion-robust^140^. Finally, haemoglobin absorption occlusion has also been highlighted as a cause for concern, as single-photon widefield imaging of green indicators is contaminated by stimulus-locked haemodynamic and intrinsic-signal changes that absorb in the green band, which is best mitigated by choosing red- or near-infra-red-shifted indicators^141^.

### Engineering opsins and indicators

Protein engineering has enabled actuators and indicators with reduced spectral crowding and enhanced kinetics. Directed evolution and rational design produce indicators with high quantum yields, faster kinetics, and red-or near-infra-red-shifted spectra that move imaging further from typical opsin activating ranges. Opsins can be engineered for narrower action spectra, lower sensitivity to imaging light, and improved localisation to the soma or other cellular compartments, reducing photostimulation from scattered photons. Mutations enhancing trafficking, reducing leak currents or shifting peak activation wavelengths further expand the design space for crosstalk-free combinations.

Beyond protein engineering, exogenous materials have also been used to enhance opsin performance. Nanostructures such as bioinspired plasmonic nanoantennas have been proposed to locally amplify light intensity for optogenetic applications, lowering the optical power supplied to the tissue required to drive integrated channelrhodopsins, leading to reduced crosstalk^142^.

### 2P imaging and stimulation

Spatial crosstalk can be mitigated by the use of 2P microscopy, such as holography and tighter optics, where the power can be reduced, and the spatial spread can be mapped empirically^6^. Additionally, imaging across large fields of view to limit dwell time per pixel will also minimise crosstalk^95^.

### Temporal and spatial multiplexing/gating

Crosstalk can also be mitigated by separating imaging and stimulation in time or space. Interleaved acquisition, where stimulation pulses are alternated with imaging frames, prevents the indicator from being exposed to stimulation during detection windows. Spatial light modulators, such as digital micromirror devices (see Fig. 4-4), can precisely gate illumination^143^. Spatial methods such as patterned illumination, holographic stimulation, or scanless optogenetics restrict photostimulation to targeted structures while imaging is performed elsewhere, reducing stray light exposure and limiting off-target activation^95^. Furthermore, fluorescence lifetime has also been shown to be of use, and scalable, as an added degree of freedom which one can employ for multiplexing^144^, though sometimes it is infeasible when utilising voltage indicators which require fast sampling rates (see Fig. 4-5). Lifetime imaging has been applied to voltage indicators in neurons^93^ and, more recently, to multiplexed in vivo functional imaging of calcium across neuronal and glial populations^145^, making lifetime separation a realistic route to reduce indicator-side crosstalk.

### Computational correction and calibration

Computational tools complement hardware-based strategies (see Fig. 4-6). Crosstalk can be modelled and subtracted using regression-based approaches^146^, blind source separation, or convolutional neural networks trained on paired imaging-stimulation datasets^147,148^. Calibration is critical for each recording site, crosstalk should be quantified by measuring fluorescence changes under imaging light alone, stimulation light alone and combined conditions^149,150^. These calibration curves enable real-time signal deconvolution and correction of stimulation-induced artefacts.

### Standardised reporting

Reproducibility requires transparent reporting. Authors should provide excitation and emission spectra for both actuator and indicators, including any spectral overlap regions. All wavelengths, irradiances, exposure durations and stimulation patterns must be stated clearly. Hardware details, including laser type, filters, dichroics and detectors, should be described comprehensively (see Fig. 4-7). Expression constructs (promoters, trafficking motifs, targeting domains), delivery methods, animal models and tissue preparations must be reported. Supplementary information should include raw data or calibration measurements quantifying (a) imaging-light effects on the actuator and (b) stimulation-light bleed-through into the detection channel. This standardisation accelerates methods comparison and supports the development of community-wide benchmarking Fig. 4 shows a graphical representation of strategies employed by Fan et al., for the purpose of minimising crosstalk^151^.

## Bidirectional optogenetics: crosstalk free combinations

### Reported crosstalk-free pairings

This all-optical approach to imaging neurons is where optogenetic activators/inhibitors, such as channel rhodopsin along with indicators like, GECIs and GEVIs are used together to non-invasively record and manipulate neuronal activity. We highlight crosstalk-free actuator and indicator combinations from literature (see Table 5) and potential candidates for future studies.

**Table 5:** Reported crosstalk in actuator-indicator pairings

Actuator	Indicator	Stimulation/Readout	Modality	Reported outcome	System	Refs.
stCoChR	jRCaMP1a	920 nm 2P holographic/1100 nm 2P readout	2P holography	stCoChR allowed neuronal stimulation with more than 10-fold lower average power and no spectral crosstalk	mouse cortex; in vivo	^32^
CheRiff	FR-GECO1a/1c	470 nm/596 nm	1P widefield	FR-GECO1a/1c exhibited Δ*F*/*F*0 values of 100% and 280%, respectively, indicating that they are well-suited for multiplexing with the blue-light-activated optogenetic actuator CheRiff	Primary rat hippocampal and cortical neurons; in vitro	^134^
CoChR	NIR-GECO2G	470 nm/640 nm	1P	the NIR readout does not activate the opsin; the combination of NIR-GECO2 and CoChR provides a robust all-optical method	C. elegans and X. laevis; in vivo	^39^
CheRiff (soma-targeted)	Voltron	488 nm/594 nm (1 P); 1135 nm (2 P)	1P and 2P	488-nm illumination did not substantially affect the GEVI performance, under either 1P or 2P voltage imaging conditions	Mouse barrel cortex; in vivo	^54^
CoChR	Archon1	470 nm/637 nm	1P	Under blue light at 4.8 mW/mm2, and with red illumination, Archons showed <2% changes in fluorescence, allowing it to be used in conjunction with CoChR	HEK293FT cells & Mouse Hippocampal neurons; in vitro	^44^
CheRiff	nirButterfly	640 nm/670 nm (donor), 720 nm (acceptor)	1P	Sufficient SNR for reliable detection of action potentials	Cortical and hippocampal neurons; in vitro	^55^
eTsChR	R-CaMP1.07	~470 nm/ ~ 1200 nm (2P)	2P holography	Red-shifted 2 P excitation raised red-GECI sensitivity	PC12h cells; in vitro	^194^
ChR2/ChrimsonR	GCaMP6s	~920 nm (2P, shared laser)	single femtosecond laser	Found that 5 mW is a nearly cross-talk-free power for both ChR2 and ChrimsonR	larval zebrafish; in vivo	^6^

Wavelengths are approximate; outcomes are as reported under the specific conditions of each study and are not directly comparable across preparations.

Experiments in mouse cortex in vivo, co-expressing the blue-light activator soma targeting opsin, stCoChR and a red-shifted calcium indicator, jRCaMP1a, combining holographic 2P stimulation of the opsin and imaging of the indicator allowed neuronal stimulation at more than 10-fold lower average power, with no detectable spectral crosstalk^32^. This pairing is instructive as a design template: soma-restricted stCoChR confines photostimulation to the somatic membrane (limiting spatial and cellular crosstalk), the blue-green opsin and the red-shifted jRCaMP1a, limiting spectral crosstalk, and two-photon holographic stimulation adds axial confinement. Another study co-expressed FR-GECO and CheRiff in cultured neurons, where CheRiff was activated by blue light at 470 nm and the response was detected by both FR-GECO1a and FR-GECO1c calcium indicators under 596 nm excitation^134^. CheRiff was stimulated for 0.6 s with 0.07 mW/mm^2^ irradiance, resulting in Δ*F*/*F*_0_ values of 20% and 60% for FR-GECO1a and FR-GECO1c, respectively^134^. The power was increased to 0.35 mW/mm^2^, resulting in an increase in Δ*F*/*F*_0_ values of 100% for FR-GECO1a and 280% for FR-GECO1c^134^. These results show that the red-shifted indicators, FR-GECO1a and FR-GECO1c, can be multiplexed with blue-light-activated optogenetic actuator CheRiff^134^ with higher power to achieve better results. Further studies in *C.elegans* neurons co-expressing CoChR blue-light activator, and the NIR-GECO2G red-light responding calcium indicator resulted in a decrease in NIR-GECO2G fluorescence from −Δ*F*/*F*_0_ 30% to 90%^39^, showing the CoChR and NIR-GECO2G together provide a crosstalk-free combination.

In mice in vivo neurons, soma localising, CheRiff, a blue activating opsin and the soma localising voltage indicator, Voltron_608_ were expressed in layer 1 barrel cortex of mice using pulses of 488 nm at 1.5 mW/mm^2^ irradiance were tested with both 2P (1135 nm, 500-Hz scan frequency, 15 to 54 mW) and 1P (594 nm, 30 mW/mm^2^) microscopy^54^. Spontaneous and stimulation spike-triggered average spike waveforms, which were similar between 1P and 2P imaging and did not affect GEVIs performance^54^. Another suitable candidate for optogenetics applications is Archon1, a voltage indicator tested with blue light intensities of 470/20 nm light pulses at 4.8 mW/mm^2^ for 500 ms at 0.5 Hz, showing <2% change in fluorescence, and the changes could be seen in neuronal processes in culture and in single dendritic spines^44^. Cultured primary mouse hippocampal neurons co-expressing CoChR an activator and Archon1 as the voltage indicator were tested using blue light illumination at 470/20 nm LED light, 10 Hz for 1 ms pulses at 0.14 mW/mm^2^ and the red excitation was using a 637 nm laser light at 1.5 W/mm^2^, imaged acquired at the soma of the neuron with image acquisition rate of 1 kHz resulted in 20% change in fluorescence due to an action potential^44^.

Another study used CheRiff co-transfected with GEVI, nirButterfly in cultured cortical neurons, with CheRiff activated by a blue band pass filter with LED light combined with a red light LED using a 600 nm long pass dichroic mirror^55^. Optical recordings showed that the CheRiff- mediated action potentials and subthreshold depolarisations were observed, with no crosstalk observed with red light excitation used for nirButterfly^55^. nirButterfly GEVI resulted in sufficient SNR for reliable detection of action potential^55^. Thus, making CheRiff and nirButterfly a reliable combination of actuator and GEVI FRET-based indicator.

## Translational applications

### Vision restoration

Optogenetics gene therapy aims to restore vision by introducing light-sensitive opsins into surviving inner-retina neurons. This has been done in blind individuals; however, recent studies show that earlier treatment, while some residual native vision remains, offers advantages^152^. Retinal degeneration affects both outer and inner retina, inducing remodelling that can impair normal signal processing, potentially limiting optogenetic outcomes; thus, earlier intervention may improve short-term function and prevent maladaptive rewiring, leading to better long-term results^153^. Moreover, restricting therapy to end-stage blindness would leave patients with years of debilitating low vision and exclude many with macular diseases such as age-related macular degeneration, who never reach complete loss of native light perception^152,154^. However, Carpentiero et al.^152^ showed interactions between native and prosthetic visual responses: ReaChR expressed in ON-bipolar cells in mice produced an electroretinography signature stronger when photoreceptors were still healthy, but native responses were dampened when ReaChR was present^152^, thus highlighting that both spectral and temporal crosstalk could disrupt photoreceptor to bipolar cell signalling.

Currently, there are several opsins in clinical trials. The first reported case of optogenetic functional recovery involved AAV-ChrimsonR delivered to a 58-year-old patient, with light stimulation via engineered goggles^155^. This ongoing PIONEER trial delivers ChrimsonR intravitreally to target ganglion cells, where a patient with inherited retinal degeneration showed recovery of light perception in low-vision tasks and light-evoked cortical responses recorded by electroencephalography^155^. A current disadvantage of ChrimsonR in retinal ganglion cells is reliance on light-amplifying goggles requiring relatively high light intensities, posing a phototoxicity risk for long-term use. A second, subtler hazard is spatial crosstalk. As stimulation light enters the eye through the pupil, it must pass through the nerve fibre layer to reach the ganglion cell somata. If the opsin localisation is not confined to the soma and/or dendrites, stimulation could result in elongated streak-like phosphenes, aligned with nerve-fibre bundles instead of focal activation. This would greatly limit the efficacy of optogenetic bionic eye implementations and reproduce the distorted percepts documented in electrical epiretinal bionic eye implants^156^.

## Challenges and Trade-offs

Optogenetics has very promising potential; however, challenges and trade-off remain a hindrance to its application in vivo and in clinical translation. Brightness is a challenge as the field is moving towards red/NIR tools, which suffer from lower quantum yield, slower kinetics, or low photostability. Power-related phototoxicity is another challenge, as longer wavelengths penetrate tissue better, but 2P stimulation requires higher power, which can heat tissue or cause damage. The complexity of hardware, where systems for 2P stimulation at long wavelengths and imaging with holography are expensive and technically demanding. From a biological perspective, expression levels and targeting of the actuator and indicator must be co-expressed well; mis-localised expression can produce unexpected activation or background leading to crosstalk. Soma-targeting actuators and indicators have emerged; however, they add complexity to experiments. Another challenge is that theoretical spectral separation can be compromised by tissue scatter, autofluorescence, or absorption, which changes effective light delivery. Temporal resolution issues can arise from gating or slower indicators reducing temporal fidelity; additionally, some bidirectional experiments require millisecond or sub-millisecond precision for optimal results.

## Conclusion

Designing and engineering more optogenetic tools is essential despite being difficult and time-consuming^22^. The tools need to be ion specific, light sensitive and have the optimal kinetics properties to expand applications of optogenetics in the future^22^. There is a need for improved red/NIR actuators and indicators with high brightness that require lower or shorter amounts of exposure to higher-power stimulation at longer wavelengths using 2P and result in minimal tissue damage. Advancements in opsins with long wavelength 2P excitation and narrow spectral profiles will allow stimulation at longer wavelengths with minimal bleed into indicator channels, therefore reducing crosstalk. Machine learning can also mitigate complexity and time-consuming constraints in designing and engineering improved optogenetic tools. Additionally, delivery of the optogenetic tools must be specific; as mentioned in the delivery of actuators and indicators section, advancements in AAV delivery and promoter specificity are required. The generation of stably expressing human cell integrating both opsin and indicator via CRISPR is also vital for advancing the field.

Delivery of the actuators into in vivo models is technically challenges due to the lack of light penetration from scattering and absorption with blue light; red-shifted rhodopsin enhances light sensitivity and tissue penetration, but more advanced optical tools are necessary for precise light delivery while minimising damage. Using machine learning to better model crosstalk and in vivo scattering will improve the prediction of tissue optics, spectral tails, scattering and absorption, enabling low-crosstalk designs. Magnetic field has been used to penetrate tissues via magnetogenetics, but kinetics are slow (seconds), and expression can be inconsistent^157^. Future development is needed for custom fibre-based systems that are more accessible for labs without full 2P or holography setups. The custom fibre-based systems need to carry more signal, be bendable to shape light waves, and have improved signal quality via a higher numerical aperture to collect more light.

Real-time computational correction and closed-loop systems will automate crosstalk correction and adaptive imaging/stimulation based on feedback, generating optimal real-time results. These improvements will expand optogenetics into other modalities and tissues such as the retina, heart, spinal cord and beyond, where optical access and scattering differ. There is strong translational potential for optogenetics in therapy and diagnosis.

All-optical, bidirectional optogenetics is increasingly feasible due to advances in actuator/reporter engineering, optics and imaging/stimulation hardware. We have shown combinations in the literature achieving low or near-negligible crosstalk, especially using spectral separation, two-photon stimulation at long wavelengths, soma-targeting opsins and red/NIR indicators. However, crosstalk remains a concern; choosing the right combination enables optimal bidirectional optogenetics applications. Looking forward, improved NIR indicators, better opsins and more accessible instrumentation and computational tools will yield robust, crosstalk-free all-optical control with broad biomedical impact. The future is bright for the field of bidirectional optogenetics, encouraging cross-collaboration with bioengineers, neuroscientists and physicists for the advancement of the field.

## Data Availability

No new data were generated in this Review. All values reported in Tables 1–5 were extracted from the primary literature cited in each table.

## Code availability

No custom code or software was generated or used in this Review.

## References

[1] Boyden, E. S., Zhang, F., Bamberg, E., Nagel, G. & Deisseroth, K. Millisecond-timescale, genetically targeted optical control of neural activity. *Nat. Neurosci.* **8**, 1263–1268 (2005). 16116447 10.1038/nn1525

[2] Piatkevich, K. D. & Boyden, E. S. Optogenetic control of neural activity: the biophysics of microbial rhodopsins in neuroscience. *Quart. Rev. Biophys.* **57**, e1 (2024). 10.1017/S0033583523000033 37831008

[3] Nagel, G. et al. Light activation of channelrhodopsin-2 in excitable cells of caenorhabditis elegans triggers rapid behavioral responses. *Curr. Biol.* **15**, 2279–2284 (2005). 16360690 10.1016/j.cub.2005.11.032

[4] Zhang, F. et al. Multimodal fast optical interrogation of neural circuitry. *Nature* **446**, 633–639 (2007). 17410168 10.1038/nature05744

[5] Deisseroth, K. Optogenetics. *Nat. Methods* **8**, 26–29 (2011). 21191368 10.1038/nmeth.f.324 PMC6814250

[6] Yan, G. et al. Active pixel power control for crosstalk-free all-optical neural interrogation. *Nat. Commun.* **17**, 3195 (2026). 41673394 10.1038/s41467-026-69419-8 PMC13057474

[7] Russell, L. E. et al. All-optical interrogation of neural circuits in behaving mice. *Nat. Protoc.* **17**, 1579–1620 (2022). 35478249 10.1038/s41596-022-00691-w PMC7616378

[8] Sridharan, S. et al. High-performance microbial opsins for spatially and temporally precise perturbations of large neuronal networks. *Neuron* **110**, 1139–1155.e6 (2022). 35120626 10.1016/j.neuron.2022.01.008 PMC8989680

[9] Chen, I.-W. et al. In vivo sub-millisecond two-photon optogenetics with temporally focused patterned light. *J. Neurosci*. 1785–18 10.1523/JNEUROSCI.1785-18.2018 (2019). 10.1523/JNEUROSCI.1785-18.2018 PMC6495136 30833505

[10] Vierock, J. et al. BiPOLES is an optogenetic tool developed for bidirectional dual-color control of neurons. *Nat. Commun.* **12**, 4527 (2021). 34312384 10.1038/s41467-021-24759-5 PMC8313717

[11] LaFosse, P. K. et al. Bicistronic expression of a high-performance calcium indicator and opsin for all-optical stimulation and imaging at cellular resolution. *eNeuro* **10**, ENEURO.0378-22.2023 (2023). 36858826 10.1523/ENEURO.0378-22.2023 PMC10062490

[12] Forli, A. et al. Two-photon bidirectional control and imaging of neuronal excitability with high spatial resolution in vivo. *Cell Rep.* **22**, 3087–3098 (2018). 29539433 10.1016/j.celrep.2018.02.063 PMC5863087

[13] Marchal, G. A. et al. Recent advances and current limitations of available technology to optically manipulate and observe cardiac electrophysiology. *Pflug. Arch. Eur. J. Physiol.* **475**, 1357–1366 (2023). 10.1007/s00424-023-02858-0 PMC10567935 37770585

[14] Mardinly, A. R. et al. Precise multimodal optical control of neural ensemble activity. *Nat. Neurosci.* **21**, 881–893 (2018). 29713079 10.1038/s41593-018-0139-8 PMC5970968

[15] Marshel, J. H. et al. Cortical layer–specific critical dynamics triggering perception. *Science* **365**, eaaw5202 (2019). 31320556 10.1126/science.aaw5202 PMC6711485

[16] Eldar, D. et al. Optogenetic approaches for neural tissue regeneration: a review of basic optogenetic principles and target cells for therapy. *Neural Regen. Res.* **21**, 521–533 (2026). 39995064 10.4103/NRR.NRR-D-24-00685 PMC12220681

[17] Nagel, G. et al. Channelrhodopsin-2, a directly light-gated cation-selective membrane channel. *Proc. Natl. Acad. Sci. USA* **100**, 13940–13945 (2003). 14615590 10.1073/pnas.1936192100 PMC283525

[18] Klapoetke, N. C. et al. Independent optical excitation of distinct neural populations. *Nat. Methods* **11**, 338–346 (2014). 24509633 10.1038/nmeth.2836 PMC3943671

[19] Yan, B. et al. A clinically viable approach to restoring visual function using optogenetic gene therapy. *Mol. Ther. Methods Clin. Dev.* **29**, 406–417 (2023). 37251979 10.1016/j.omtm.2023.05.005 PMC10213293

[20] Schobert, B. & Lanyi, J. K. Halorhodopsin is a light-driven chloride pump. *J. Biol. Chem.* **257**, 10306–10313 (1982). 7107607

[21] Chow, B. Y. et al. High-performance genetically targetable optical neural silencing by light-driven proton pumps. *Nature* **463**, 98–102 (2010). 20054397 10.1038/nature08652 PMC2939492

[22] Duan, X., Zhu, M. & Gao, S. Two decades of optogenetic tools: a retrospective and a look ahead. *Adv. Genet.* **6**, e00021 (2025). 41036479 10.1002/ggn2.202500021 PMC12482941

[23] Broser, M. et al. NeoR, a near-infrared absorbing rhodopsin. *Nat. Commun.* **11**, 5682 (2020). 33173168 10.1038/s41467-020-19375-8 PMC7655827

[24] Broser, M. et al. Diversity of rhodopsin cyclases in zoospore-forming fungi. *Proc. Natl. Acad. Sci. USA* **120**, e2310600120 (2023). 37871207 10.1073/pnas.2310600120 PMC10622942

[25] Palombo, R. et al. Retinal chromophore charge delocalization and confinement explain the extreme photophysics of neorhodopsin. *Nat. Commun.* **13**, 6652 (2022). 36333283 10.1038/s41467-022-33953-y PMC9636224

[26] Van Stokkum, I. H. M. et al. Retinal to retinal energy transfer in a bistable microbial rhodopsin dimer. *J. Am. Chem. Soc.* **147**, 14468–14480 (2025). 40245178 10.1021/jacs.5c01276 PMC12046560

[27] Xu, C., Nedergaard, M., Fowell, D. J., Friedl, P. & Ji, N. Multiphoton fluorescence microscopy for in vivo imaging. *Cell* **187**, 4458–4487 (2024). 39178829 10.1016/j.cell.2024.07.036 PMC11373887

[28] Shemesh, O. A. et al. Temporally precise single-cell-resolution optogenetics. *Nat. Neurosci.* **20**, 1796–1806 (2017). 29184208 10.1038/s41593-017-0018-8 PMC5726564

[29] Nikolenko, V. SLM microscopy: scanless two-photon imaging and photostimulation using spatial light modulators. *Front. Neural Circuits* **2**, 5 (2008). 10.3389/neuro.04.005.2008 PMC2614319 19129923

[30] Papagiakoumou, E., De Sars, V., Oron, D. & Emiliani, V. Patterned two-photon illumination by spatiotemporal shaping of ultrashort pulses. *Opt. Express* **16**, 22039 (2008). 19104638 10.1364/oe.16.022039

[31] Dayan, E., Censor, N., Buch, E. R., Sandrini, M. & Cohen, L. G. Noninvasive brain stimulation: from physiology to network dynamics and back. *Nat. Neurosci.* **16**, 838–844 (2013). 23799477 10.1038/nn.3422 PMC4876726

[32] Forli, A., Pisoni, M., Printz, Y., Yizhar, O. & Fellin, T. Optogenetic strategies for high-efficiency all-optical interrogation using blue-light-sensitive opsins. *eLife* **10**, e63359 (2021). 34032211 10.7554/eLife.63359 PMC8177884

[33] Milosevic, M. M., Jang, J., McKimm, E. J., Zhu, M. H. & Antic, S. D. In vitro testing of voltage indicators: archon1, arclightd, asap1, asap2s, asap3b, bongwoori-pos6, berst1, flicr1, and chi-vsfp-butterfly. *eNeuro* **7**, ENEURO.0060-20.2020 (2020). 32817120 10.1523/ENEURO.0060-20.2020 PMC7540930

[34] Zhang, Y. et al. Fast and sensitive GCaMP calcium indicators for imaging neural populations. *Nature* **615**, 884–891 (2023). 36922596 10.1038/s41586-023-05828-9 PMC10060165

[35] Crivici, A. & Ikura, M. Molecular and structural basis of target recognition by calmodulin. *Annu. Rev. Biophys. Biomol. Struct.* **24**, 85–116 (1995). 7663132 10.1146/annurev.bb.24.060195.000505

[36] Chen, T.-W. et al. Ultrasensitive fluorescent proteins for imaging neuronal activity. *Nature* **499**, 295–300 (2013). 23868258 10.1038/nature12354 PMC3777791

[37] Chai, F. et al. Development of a mirfp680-based fluorescent calcium ion biosensor using end-optimized transposons. *ACS Sens.* **9**, 3394–3402 (2024). 38822813 10.1021/acssensors.4c00727 PMC11218748

[38] Qian, Y. et al. A genetically encoded near-infrared fluorescent calcium ion indicator. *Nat. Methods* **16**, 171–174 (2019). 30664778 10.1038/s41592-018-0294-6 PMC6393164

[39] Qian, Y. et al. Improved genetically encoded near-infrared fluorescent calcium ion indicators for in vivo imaging. *PLoS Biol.* **18**, e3000965 (2020). 33232322 10.1371/journal.pbio.3000965 PMC7723245

[40] Smetters, D., Majewska, A. & Yuste, R. Detecting action potentials in neuronal populations with calcium imaging. *Methods* **18**, 215–221 (1999). 10356353 10.1006/meth.1999.0774

[41] Neher, E. Usefulness and limitations of linear approximations to the understanding of Ca++ signals. *Cell Calcium* **24**, 345–357 (1998). 10091004 10.1016/s0143-4160(98)90058-6

[42] Peterka, D. S., Takahashi, H. & Yuste, R. Imaging voltage in neurons. *Neuron* **69**, 9–21 (2011). 21220095 10.1016/j.neuron.2010.12.010 PMC3387979

[43] Lin, M. Z. & Schnitzer, M. J. Genetically encoded indicators of neuronal activity. *Nat. Neurosci.* **19**, 1142–1153 (2016). 27571193 10.1038/nn.4359 PMC5557009

[44] Piatkevich, K. D. et al. A robotic multidimensional directed evolution approach applied to fluorescent voltage reporters. *Nat. Chem. Biol.* **14**, 352–360 (2018). 29483642 10.1038/s41589-018-0004-9 PMC5866759

[45] Bayraktar, H. et al. Ultrasensitive measurements of microbial rhodopsin photocycles using photochromic fret. *Photochem. Photobiol.* **88**, 90–97 (2012). 22010969 10.1111/j.1751-1097.2011.01011.x PMC3253248

[46] Gong, Y., Wagner, M. J., Zhong Li, J. & Schnitzer, M. J. Imaging neural spiking in brain tissue using fret-opsin protein voltage sensors. *Nat. Commun.* **5**, 3674 (2014). 24755708 10.1038/ncomms4674 PMC4247277

[47] Zou, P. et al. Bright and fast multicoloured voltage reporters via electrochromic fret. *Nat. Commun.* **5**, 4625 (2014). 25118186 10.1038/ncomms5625 PMC4134104

[48] Gong, Y. et al. High-speed recording of neural spikes in awake mice and flies with a fluorescent voltage sensor. *Science* **350**, 1361–1366 (2015). 26586188 10.1126/science.aab0810 PMC4904846

[49] Kannan, M. et al. Dual-polarity voltage imaging of the concurrent dynamics of multiple neuron types. *Science* **378**, eabm8797 (2022). 36378956 10.1126/science.abm8797 PMC9703638

[50] Fan, C.-H. et al. Selective activation of cells by piezoelectric molybdenum disulfide nanosheets with focused ultrasound. *ACS Nano* **17**, 9140–9154 (2023). 37163347 10.1021/acsnano.2c12438

[51] Abdelfattah, A. S. et al. Sensitivity optimization of a rhodopsin-based fluorescent voltage indicator. *Neuron* **111**, 1547–1563.e9 (2023). 37015225 10.1016/j.neuron.2023.03.009 PMC10280807

[52] Abdelfattah, A. S. et al. Bright and photostable chemigenetic indicators for extended in vivo voltage imaging. *Science* **365**, 699–704 (2019). 31371562 10.1126/science.aav6416

[53] Liu, S. et al. A far-red hybrid voltage indicator enabled by bioorthogonal engineering of rhodopsin on live neurons. *Nat. Chem.* **13**, 472–479 (2021). 33859392 10.1038/s41557-021-00641-1

[54] Brooks, F. P. et al. Photophysics-informed two-photon voltage imaging using fret-opsin voltage indicators. *Sci. Adv.* **11**, eadp5763 (2025). 39772682 10.1126/sciadv.adp5763 PMC11708879

[55] Monakhov, M. V. et al. Screening and cellular characterization of genetically encoded voltage indicators based on near-infrared fluorescent proteins. *ACS Chem. Neurosci.* **11**, 3523–3531 (2020). 33063984 10.1021/acschemneuro.0c00046

[56] Xiao, X. et al. Engineering of genetically encoded bright near-infrared fluorescent voltage indicator. *IJMS* **26**, 1442 (2025). 40003908 10.3390/ijms26041442 PMC11855178

[57] Sigworth, F. J. & Neher, E. Single na+ channel currents observed in cultured rat muscle cells. *Nature* **287**, 447–449 (1980). 6253802 10.1038/287447a0

[58] Hamill, O. P., Marty, A., Neher, E., Sakmann, B. & Sigworth, F. J. Improved patch-clamp techniques for high-resolution current recording from cells and cell-free membrane patches. *Pflug. Arch. Eur. J. Physiol.* **391**, 85–100 (1981). 10.1007/BF00656997 6270629

[59] Guru, A., Post, R. J., Ho, Y.-Y. & Warden, M. R. Making sense of optogenetics. *IJNPPY* **18**, pyv079 (2015). 10.1093/ijnp/pyv079 PMC4756725 26209858

[60] Bulcha, J. T., Wang, Y., Ma, H., Tai, P. W. L. & Gao, G. Viral vector platforms within the gene therapy landscape. *Signal Transduct. Target. Ther.* **6**, 53 (2021). 33558455 10.1038/s41392-021-00487-6 PMC7868676

[61] Kubota, S. et al. Optogenetic recruitment of spinal reflex pathways from large-diameter primary afferents in non-transgenic rats transduced with aav9/channelrhodopsin 2. *J. Physiol.* **597**, 5025–5040 (2019). 31397900 10.1113/JP278292 PMC6851594

[62] Tong, Q. et al. D1 receptor-expressing neurons in ventral tegmental area alleviate mouse anxiety-like behaviors via glutamatergic projection to lateral septum. *Mol. Psychiatry* **28**, 625–638 (2023). 36195641 10.1038/s41380-022-01809-y PMC9531220

[63] Hudry, E. & Vandenberghe, L. H. Therapeutic AAV gene transfer to the nervous system: a clinical reality. *Neuron* **101**, 839–862 (2019). 30844402 10.1016/j.neuron.2019.02.017 PMC11804970

[64] Stroh, A. et al. Tracking stem cell differentiation in the setting of automated optogenetic stimulation. *Stem Cells* **29**, 78–88 (2011). 21280159 10.1002/stem.558 PMC4182948

[65] Zhang, F. et al. Optogenetic interrogation of neural circuits: technology for probing mammalian brain structures. *Nat. Protoc.* **5**, 439–456 (2010). 20203662 10.1038/nprot.2009.226 PMC4503465

[66] Wiegert, J. S., Gee, C. E. & Oertner, T. G. Single-cell electroporation of neurons. *Cold Spring Harb. Protoc*. **2017**, pdb.prot094904 (2017). 10.1101/pdb.prot094904 28148856

[67] Gonzalez, K. C. et al. Visually guided in vivo single-cell electroporation for monitoring and manipulating mammalian hippocampal neurons. *Nat. Protoc.* **20**, 1468–1484 (2025). 39930062 10.1038/s41596-024-01099-4 PMC12151752

[68] Zou, M., De Koninck, P., Neve, R. L. & Friedrich, R. W. Fast gene transfer into the adult zebrafish brain by herpes simplex virus 1 (hsv-1) and electroporation: methods and optogenetic applications. *Front. Neural Circuits* **8**, 41 (2014). 10.3389/fncir.2014.00041 PMC4018551 24834028

[69] Dhakal, K., Batabyal, S., Wright, W., Kim, Y. & Mohanty, S. Optical delivery of multiple opsin-encoding genes leads to targeted expression and white-light activation. *Light Sci. Appl.* **4**, e352 (2015).

[70] Jia, Z. et al. Stimulating cardiac muscle by light: cardiac optogenetics by cell delivery. *Circ. Arrhythm. Electrophysiol.* **4**, 753–760 (2011). 21828312 10.1161/CIRCEP.111.964247 PMC3209525

[71] Chakravarty, P., Qian, W., El-Sayed, M. A. & Prausnitz, M. R. Delivery of molecules into cells using carbon nanoparticles activated by femtosecond laser pulses. *Nat. Nanotechnol.* **5**, 607–611 (2010). 20639882 10.1038/nnano.2010.126 PMC2917490

[72] Lukianova-Hleb, E. Y., Mutonga, M. B. G. & Lapotko, D. O. Cell-specific multifunctional processing of heterogeneous cell systems in a single laser pulse treatment. *ACS Nano* **6**, 10973–10981 (2012). 23167546 10.1021/nn3045243 PMC3528843

[73] Inagaki, H. K. et al. Optogenetic control of drosophila using a red-shifted channelrhodopsin reveals experience-dependent influences on courtship. *Nat. Methods* **11**, 325–332 (2014). 24363022 10.1038/nmeth.2765 PMC4151318

[74] Hsiao, P.-Y. et al. Non-invasive manipulation of drosophila behavior by two-photon excited red-activatable channelrhodopsin. *Biomed. Opt. Express* **6**, 4344 (2015). 26601000 10.1364/BOE.6.004344 PMC4646544

[75] Madisen, L. et al. A toolbox of cre-dependent optogenetic transgenic mice for light-induced activation and silencing. *Nat. Neurosci.* **15**, 793–802 (2012). 22446880 10.1038/nn.3078 PMC3337962

[76] Ting, J. T. & Feng, G. Development of transgenic animals for optogenetic manipulation of mammalian nervous system function: progress and prospects for behavioral neuroscience. *Behav. Brain Res.* **255**, 3–18 (2013). 23473879 10.1016/j.bbr.2013.02.037 PMC4030747

[77] Zhou, Y. et al. A rapid and efficient red-light-activated cre recombinase system for genome engineering in mammalian cells and transgenic mice. *Nucleic Acids Res.* **53**, gkaf758 (2025). 40795961 10.1093/nar/gkaf758 PMC12342929

[78] Kuwasaki, Y. et al. A red light–responsive photoswitch for deep tissue optogenetics. *Nat. Biotechnol.* **40**, 1672–1679 (2022). 35697806 10.1038/s41587-022-01351-w

[79] Zhou, Y. et al. A small and highly sensitive red/far-red optogenetic switch for applications in mammals. *Nat. Biotechnol.* **40**, 262–272 (2022). 34608325 10.1038/s41587-021-01036-w

[80] Chenouard, V. et al. Advances in genome editing and application to the generation of genetically modified rat models. *Front. Genet.* **12**, 615491 (2021). 33959146 10.3389/fgene.2021.615491 PMC8093876

[81] Feng, G. et al. Opportunities and limitations of genetically modified nonhuman primate models for neuroscience research. *Proc. Natl. Acad. Sci. USA* **117**, 24022–24031 (2020). 32817435 10.1073/pnas.2006515117 PMC7533691

[82] Ben-Simon, Y. et al. A suite of enhancer AAVs and transgenic mouse lines for genetic access to cortical cell types. *Cell***188**, 3045–3064.e23 (2025).40403729 10.1016/j.cell.2025.05.002

[83] Aline Schmoll, K. et al. Genome engineering of a neuronal specific, optogenetic, induced pluripotent stem cell line. *Stem Cell Res.* **75**, 103317 (2024). 38295750 10.1016/j.scr.2024.103317

[84] Léger-Charnay, E., Slembrouck-Brec, A. & Goureau, O. Engineering specific human iPS reporter cell lines to generate optogenetically modified photoreceptors. in *Retinal Degenerative Diseases XX* (eds. Bowes Rickman, C. et al.) vol. 1468 409–414 (Springer Nature Switzerland, 2025). 10.1007/978-3-031-76550-6_67 39930230

[85] Li, R. et al. Advances in two-photon imaging for monitoring neural activity in behaving mice. *Front. Neurosci.* **19**, 1597151 (2025). 40860843 10.3389/fnins.2025.1597151 PMC12375630

[86] Denk, W., Strickler, J. H. & Webb, W. W. Two-photon laser scanning fluorescence microscopy. *Science* **248**, 73–76 (1990). 2321027 10.1126/science.2321027

[87] Horton, N. G. et al. In vivo three-photon microscopy of subcortical structures within an intact mouse brain. *Nat. Photon* **7**, 205–209 (2013). 10.1038/nphoton.2012.336 PMC3864872 24353743

[88] Akbari, N., Rebec, M. R., Xia, F. & Xu, C. Imaging deeper than the transport mean free path with multiphoton microscopy. *Biomed. Opt. Express* **13**, 452 (2022). 35154884 10.1364/BOE.444696 PMC8803047

[89] Liu, H. et al. In vivo deep-brain structural and hemodynamic multiphoton microscopy enabled by quantum dots. *Nano Lett.* **19**, 5260–5265 (2019). 31268725 10.1021/acs.nanolett.9b01708

[90] Chien, M.-P. et al. Photoactivated voltage imaging in tissue with an archaerhodopsin-derived reporter. *Sci. Adv.* **7**, eabe3216 (2021). 33952514 10.1126/sciadv.abe3216 PMC8099184

[91] Liu, Z. et al. Sustained deep-tissue voltage recording using a fast indicator evolved for two-photon microscopy. *Cell* **185**, 3408–3425.e29 (2022). 35985322 10.1016/j.cell.2022.07.013 PMC9563101

[92] Chamberland, S. et al. Fast two-photon imaging of subcellular voltage dynamics in neuronal tissue with genetically encoded indicators. *eLife* **6**, e25690 (2017). 28749338 10.7554/eLife.25690 PMC5584994

[93] Brinks, D., Klein, A. J. & Cohen, A. E. Two-photon lifetime imaging of voltage indicating proteins as a probe of absolute membrane voltage. *Biophys. J.* **109**, 914–921 (2015). 26331249 10.1016/j.bpj.2015.07.038 PMC4564826

[94] Bando, Y., Sakamoto, M., Kim, S., Ayzenshtat, I. & Yuste, R. Comparative evaluation of genetically encoded voltage indicators. *Cell Rep.* **26**, 802–813.e4 (2019). 30650368 10.1016/j.celrep.2018.12.088 PMC7075032

[95] Telliez, C. et al. Multiphoton neurophotonics: recent advances in imaging and manipulating neuronal circuits. *ACS Photonics* **12**, 3296–3318 (2025). 40693197 10.1021/acsphotonics.4c02101 PMC12279007

[96] Abdeladim, L., Jagadisan, U. K., Shin, H., Ogando, M. B. & Adesnik, H. Probing inter-areal computations with a cellular resolution two-photon holographic mesoscope. Preprint at 10.1101/2023.03.02.530875 (2023). 10.1038/s41593-026-02350-9 PMC13433287 42547821

[97] Xu, S. et al. Illuminating the brain: advances and perspectives in optoelectronics for neural activity monitoring and modulation. *Adv. Mater.* **35**, 2303267 (2023). 10.1002/adma.202303267 37726261

[98] Rossi, M. A. et al. A wirelessly controlled implantable led system for deep brain optogenetic stimulation. *Front. Integr. Neurosci*. **9**, 8 (2015). 10.3389/fnint.2015.00008 PMC4322607 25713516

[99] Fan, B. & Li, W. Miniaturized optogenetic neural implants: a review. *Lab Chip* **15**, 3838–3855 (2015). 26308721 10.1039/c5lc00588d

[100] Samineni, V. K. et al. Fully implantable, battery-free wireless optoelectronic devices for spinal optogenetics. *Pain* **158**, 2108–2116 (2017). 28700536 10.1097/j.pain.0000000000000968 PMC5640477

[101] McCall, J. G. et al. Fabrication and application of flexible, multimodal light-emitting devices for wireless optogenetics. *Nat. Protoc.* **8**, 2413–2428 (2013). 24202555 10.1038/nprot.2013.158 PMC4005292

[102] Ausra, J. et al. Wireless, battery-free, subdermally implantable platforms for transcranial and long-range optogenetics in freely moving animals. *Proc. Natl. Acad. Sci. USA* **118**, e2025775118 (2021). 34301889 10.1073/pnas.2025775118 PMC8325245

[103] Kathe, C. et al. Wireless closed-loop optogenetics across the entire dorsoventral spinal cord in mice. *Nat. Biotechnol.* **40**, 198–208 (2022). 34580478 10.1038/s41587-021-01019-x PMC7612390

[104] Wu, C., Liu, X. & Ying, Y. Soft and stretchable optical waveguide: light delivery and manipulation at complex biointerfaces creating unique windows for on-body sensing. *ACS Sens.* **6**, 1446–1460 (2021). 33611914 10.1021/acssensors.0c02566

[105] Zorzos, A. N., Scholvin, J., Boyden, E. S. & Fonstad, C. G. Three-dimensional multiwaveguide probe array for light delivery to distributed brain circuits. *Opt. Lett.* **37**, 4841 (2012). 23202064 10.1364/OL.37.004841 PMC3572236

[106] Zhang, J. et al. A microelectrode array incorporating an optical waveguide device for stimulation and spatiotemporal electrical recording of neural activity. in *Proc*. *2009 Annual International Conference of the IEEE Engineering in Medicine and Biology Society* 2046–2049. 10.1109/IEMBS.2009.5333947 (IEEE, 2009). 10.1109/IEMBS.2009.5333947 19964571

[107] Wu, F. et al. An implantable neural probe with monolithically integrated dielectric waveguide and recording electrodes for optogenetics applications. *J. Neural Eng.* **10**, 056012 (2013). 23985803 10.1088/1741-2560/10/5/056012 PMC4056669

[108] Yamagiwa, S., Ishida, M. & Kawano, T. Flexible optrode array: parylene-film waveguide arrays with microelectrodes for optogenetics. In *2015 Transducers – 18th International Conference on Solid-State Sensors, Actuators and Microsystems (TRANSDUCERS)* 277–280 10.1109/TRANSDUCERS.2015.7180915 (IEEE, 2015).

[109] Clark, A. M. et al. An optrode array for spatiotemporally-precise large-scale optogenetic stimulation of deep cortical layers in non-human primates. *Commun. Biol.* **7**, 329 (2024). 38485764 10.1038/s42003-024-05984-2 PMC10940688

[110] Xu, Y. et al. Sapphire-based optrode for low noise neural recording and optogenetic manipulation. *ACS Chem. Neurosci.* **16**, 628–641 (2025). 39910858 10.1021/acschemneuro.4c00602 PMC12066107

[111] McAlinden, N. In vivo optogenetics using a utah optrode array with enhanced light output and spatial selectivity. *J. Neural Eng.* **21**, 046051 (2024). 10.1088/1741-2552/ad69c3 PMC12123438 39084245

[112] Ozden, I. et al. A coaxial optrode as multifunction write-read probe for optogenetic studies in non-human primates. *J. Neurosci. Methods* **219**, 142–154 (2013). 23867081 10.1016/j.jneumeth.2013.06.011 PMC3789534

[113] Sukesan, R. et al. 3D-printed optogenetic neural probe integrated with microfluidic tube for opsin/drug delivery. *Sci. Rep.* **15**, 28863 (2025). 40775005 10.1038/s41598-025-13654-4 PMC12331985

[114] Hososhima, S. et al. Near-infrared (nir) up-conversion optogenetics. *Sci. Rep.* **5**, 16533 (2015). 26552717 10.1038/srep16533 PMC4639720

[115] Shah, S. et al. Hybrid upconversion nanomaterials for optogenetic neuronal control. *Nanoscale* **7**, 16571–16577 (2015). 26415758 10.1039/c5nr03411f PMC4712042

[116] Wu, X. et al. Dye-sensitized core/active shell upconversion nanoparticles for optogenetics and bioimaging applications. *ACS Nano* **10**, 1060–1066 (2016). 26736013 10.1021/acsnano.5b06383 PMC4913696

[117] Bansal, A., Liu, H., Jayakumar, M. K. G., Andersson-Engels, S. & Zhang, Y. Quasi-Continuous wave near-infrared excitation of upconversion nanoparticles for optogenetic manipulation of *C. elegans*. *Small* **12**, 1732–1743 (2016). 26849846 10.1002/smll.201503792

[118] Ai, X. et al. Remote regulation of membrane channel activity by site-specific localization of lanthanide-doped upconversion nanocrystals. *Angew. Chem. Int. Ed.* **56**, 3031–3035 (2017). 10.1002/anie.201612142 28157258

[119] Chen, S. et al. Near-infrared deep brain stimulation via upconversion nanoparticle–mediated optogenetics. *Science* **359**, 679–684 (2018). 29439241 10.1126/science.aaq1144

[120] Wen, S. et al. Future and challenges for hybrid upconversion nanosystems. *Nat. Photonics* **13**, 828–838 (2019).

[121] Owen, S. F., Liu, M. H. & Kreitzer, A. C. Thermal constraints on in vivo optogenetic manipulations. *Nat. Neurosci.* **22**, 1061–1065 (2019). 31209378 10.1038/s41593-019-0422-3 PMC6592769

[122] Du, Z. et al. Photoacoustic: a versatile nongenetic method for high-precision neuromodulation. *Acc. Chem. Res.* **57**, 1595–1607 (2024). 38759211 10.1021/acs.accounts.4c00119 PMC11154953

[123] Jiang, Y. et al. Neural stimulation in vitro and in vivo by photoacoustic nanotransducers. *Matter* **4**, 654–674 (2021).

[124] Wang, L. V. & Hu, S. Photoacoustic tomography: in vivo imaging from organelles to organs. *Science* **335**, 1458–1462 (2012). 22442475 10.1126/science.1216210 PMC3322413

[125] Shaykevich, S. F. et al. Multimodal fluorescence-optoacoustic in vivo imaging of the near-infrared calcium ion indicator nir-geco2g. *Photoacoustics* **41**, 100671 (2025). 39811063 10.1016/j.pacs.2024.100671 PMC11732225

[126] Kasatkina, L. A. et al. Deep-tissue high-sensitivity multimodal imaging and optogenetic manipulation enabled by biliverdin reductase knockout. *Nat. Commun.* **16**, 6469 (2025). 40659617 10.1038/s41467-025-61532-4 PMC12260061

[127] Yue, K. et al. Magneto-electric nano-particles for non-invasive brain stimulation. *PLoS ONE* **7**, e44040 (2012). 22957042 10.1371/journal.pone.0044040 PMC3434207

[128] Guduru, R. et al. Magnetoelectric ‘spin’ on stimulating the brain. *Nanomedicine* **10**, 2051–2061 (2015). 25953069 10.2217/nnm.15.52 PMC4910966

[129] Signorelli, L. et al. Biocompatible pvdf nanofibers with embedded magnetite nanodiscs enable wireless magnetoelectric stimulation in premotor cortex. *Adv. Healthc. Mater.* **14**, e03082 (2025). 40776441 10.1002/adhm.202503082 PMC12716173

[130] Abramson, J. et al. Accurate structure prediction of biomolecular interactions with alphafold 3. *Nature* **630**, 493–500 (2024). 38718835 10.1038/s41586-024-07487-w PMC11168924

[131] Shaner, N. C. et al. A bright monomeric green fluorescent protein derived from branchiostoma lanceolatum. *Nat. Methods* **10**, 407–409 (2013). 23524392 10.1038/nmeth.2413 PMC3811051

[132] Subach, O. M. et al. Novel genetically encoded bright positive calcium indicator ncamp7 based on the mneongreen fluorescent protein. *IJMS* **21**, 1644 (2020). 32121243 10.3390/ijms21051644 PMC7084697

[133] Li, J. et al. Engineering of nemo as calcium indicators with large dynamics and high sensitivity. *Nat. Methods* **20**, 918–924 (2023). 37081094 10.1038/s41592-023-01852-9 PMC10250196

[134] Dalangin, R. et al. Far-red fluorescent genetically encoded calcium ion indicators. *Nat. Commun.* **16**, 3318 (2025). 40195305 10.1038/s41467-025-58485-z PMC11977202

[135] Zimmermann, T., Rietdorf, J. & Pepperkok, R. Spectral imaging and its applications in live cell microscopy. *FEBS Lett.* **546**, 87–92 (2003). 12829241 10.1016/s0014-5793(03)00521-0

[136] Pnevmatikakis, E. A. et al. Simultaneous denoising, deconvolution, and demixing of calcium imaging data. *Neuron* **89**, 285–299 (2016). 26774160 10.1016/j.neuron.2015.11.037 PMC4881387

[137] Deneux, T. et al. Accurate spike estimation from noisy calcium signals for ultrafast three-dimensional imaging of large neuronal populations in vivo. *Nat. Commun.* **7**, 12190 (2016). 27432255 10.1038/ncomms12190 PMC4960309

[138] Friedrich, J., Zhou, P. & Paninski, L. Fast online deconvolution of calcium imaging data. *PLoS Comput. Biol.* **13**, e1005423 (2017). 28291787 10.1371/journal.pcbi.1005423 PMC5370160

[139] Shibue, R. & Komaki, F. Deconvolution of calcium imaging data using marked point processes. *PLoS Comput. Biol.* **16**, e1007650 (2020). 32163407 10.1371/journal.pcbi.1007650 PMC7093033

[140] Villette, V. et al. Ultrafast two-photon imaging of a high-gain voltage indicator in awake behaving mice. *Cell* **179**, 1590–1608.e23 (2019). 31835034 10.1016/j.cell.2019.11.004 PMC6941988

[141] Yogesh, B., Heindorf, M., Jordan, R. & Keller, G. B. Quantification of the effect of hemodynamic occlusion in two-photon imaging of mouse cortex. *eLife* **14**, RP104914 (2025). 40434064 10.7554/eLife.104914 PMC12119086

[142] Keck, C. H. C., Rommelfanger, N. J., Ou, Z. & Hong, G. Bioinspired nanoantennas for opsin sensitization in optogenetic applications: a theoretical investigation. *Multifunct. Mater.* **4**, 024002 (2021). 41694712 10.1088/2399-7532/abf0f9 PMC12900125

[143] Rullan, M., Benzinger, D., Schmidt, G. W., Milias-Argeitis, A. & Khammash, M. An optogenetic platform for real-time, single-cell interrogation of stochastic transcriptional regulation. *Mol. Cell* **70**, 745–756.e6 (2018). 29775585 10.1016/j.molcel.2018.04.012 PMC5971206

[144] Hwang, W., Raymond, T., McPartland, T., Jeong, S. & Evans, C. L. Fluorescence lifetime multiplexing (flex) for simultaneous high dimensional spatial biology in 3d. *Commun. Biol.* **7**, 1012 (2024). 39154126 10.1038/s42003-024-06702-8 PMC11330493

[145] Farrants, H. et al. A modular chemigenetic calcium indicator for multiplexed in vivo functional imaging. *Nat. Methods* **21**, 1916–1925 (2024). 39304767 10.1038/s41592-024-02411-6 PMC11466818

[146] Antin, B. et al. Removing direct photocurrent artifacts in optogenetic connectivity mapping data via constrained matrix factorization. *PLoS Comput. Biol.* **20**, e1012053 (2024). 38709828 10.1371/journal.pcbi.1012053 PMC11098512

[147] Schroff, P., Haller, E., Kuhr, S. & La Rooij, A. Rapid stochastic spatial light modulator calibration and pixel crosstalk optimization. *Opt. Express* **32**, 48957 (2024). 39876187 10.1364/OE.539548

[148] Breakstone, M. et al. Multi-frequency interpolation x-talk removal algorithm: enabling combinations of concurrent optogenetics and lock-in amplification fiber photometry via removal of optogenetic stimulation crosstalk. *ACS Chem. Neurosci.* **16**, 1694–1709 (2025). 40228799 10.1021/acschemneuro.4c00632 PMC12063611

[149] Chen, J. L., Voigt, F. F., Javadzadeh, M., Krueppel, R. & Helmchen, F. Long-range population dynamics of anatomically defined neocortical networks. *eLife* **5**, e14679 (2016). 27218452 10.7554/eLife.14679 PMC4929001

[150] Liu, C., Hao, Y., Lei, B., Zhong, Y. & Kong, L. Removing crosstalk signals in neuron activity by time multiplexed excitations in a two-photon all-optical physiology system. *Biomed. Opt. Express* **15**, 2708 (2024). 38633062 10.1364/BOE.521047 PMC11019693

[151] Fan, L. Z. et al. All-optical electrophysiology reveals the role of lateral inhibition in sensory processing in cortical layer 1. *Cell* **180**, 521–535.e18 (2020). 31978320 10.1016/j.cell.2020.01.001 PMC7259440

[152] Carpentiero, E. et al. Interaction between native and prosthetic visual responses in optogenetic visual restoration. *JCI Insight* **10**, e190785 (2025). 40232851 10.1172/jci.insight.190785 PMC12220941

[153] Pfeiffer, R. L., Marc, R. E. & Jones, B. W. Persistent remodeling and neurodegeneration in late-stage retinal degeneration. *Prog. Retin Eye Res.* **74**, 100771 (2020). 31356876 10.1016/j.preteyeres.2019.07.004 PMC6982593

[154] Cross, N., van Steen, C., Zegaoui, Y., Satherley, A. & Angelillo, L. Retinitis pigmentosa: burden of disease and current unmet needs. *Clin. Ophthalmol.* **16**, 1993–2010 (2022). 35757022 10.2147/OPTH.S365486 PMC9232096

[155] Sahel, J.-A. et al. Partial recovery of visual function in a blind patient after optogenetic therapy. *Nat. Med.* **27**, 1223–1229 (2021). 34031601 10.1038/s41591-021-01351-4

[156] Beyeler, M. et al. A model of ganglion axon pathways accounts for percepts elicited by retinal implants. *Sci. Rep.* **9**, 9199 (2019). 31235711 10.1038/s41598-019-45416-4 PMC6591412

[157] Wheeler, M. A. et al. Genetically targeted magnetic control of the nervous system. *Nat. Neurosci.* **19**, 756–761 (2016). 26950006 10.1038/nn.4265 PMC4846560

[158] Lin, J. Y., Lin, M. Z., Steinbach, P. & Tsien, R. Y. Characterization of engineered channelrhodopsin variants with improved properties and kinetics. *Biophys. J.* **96**, 1803–1814 (2009). 19254539 10.1016/j.bpj.2008.11.034 PMC2717302

[159] Hochbaum, D. R. et al. All-optical electrophysiology in mammalian neurons using engineered microbial rhodopsins. *Nat. Methods* **11**, 825–833 (2014). 24952910 10.1038/nmeth.3000 PMC4117813

[160] Mattis, J. et al. Principles for applying optogenetic tools derived from direct comparative analysis of microbial opsins. *Nat. Methods* **9**, 159–172 (2012). 10.1038/nmeth.1808 PMC4165888 22179551

[161] Gunaydin, L. A. et al. Ultrafast optogenetic control. *Nat. Neurosci.* **13**, 387–392 (2010). 20081849 10.1038/nn.2495

[162] Kleinlogel, S. et al. Ultra light-sensitive and fast neuronal activation with the ca2+-permeable channelrhodopsin catch. *Nat. Neurosci.* **14**, 513–518 (2011). 21399632 10.1038/nn.2776

[163] Alekseev, A. et al. Efficient and sustained optogenetic control of sensory and cardiac systems. *Nat. Biomed. Eng.* **10**, 277–292 (2025). 40721511 10.1038/s41551-025-01461-1 PMC12920086

[164] Rein, M. L. & Deussing, J. M. The optogenetic (r)evolution. *Mol. Genet. Genom.* **287**, 95–109 (2012). 10.1007/s00438-011-0663-7 PMC3266495 22183142

[165] Zhang, F. et al. Red-shifted optogenetic excitation: a tool for fast neural control derived from volvox carteri. *Nat. Neurosci.* **11**, 631–633 (2008). 18432196 10.1038/nn.2120 PMC2692303

[166] Lin, J. Y., Knutsen, P. M., Muller, A., Kleinfeld, D. & Tsien, R. Y. ReaChR: a red-shifted variant of channelrhodopsin enables deep transcranial optogenetic excitation. *Nat. Neurosci.* **16**, 1499–1508 (2013). 23995068 10.1038/nn.3502 PMC3793847

[167] Mager, T. et al. High frequency neural spiking and auditory signaling by ultrafast red-shifted optogenetics. *Nat. Commun.* **9**, 1750 (2018). 29717130 10.1038/s41467-018-04146-3 PMC5931537

[168] Zhao, S. et al. Improved expression of halorhodopsin for light-induced silencing of neuronal activity. *Brain Cell Biol.* **36**, 141–154 (2008). 18931914 10.1007/s11068-008-9034-7 PMC3057022

[169] Gradinaru, V. et al. Molecular and cellular approaches for diversifying and extending optogenetics. *Cell* **141**, 154–165 (2010). 20303157 10.1016/j.cell.2010.02.037 PMC4160532

[170] Chuong, A. S. et al. Noninvasive optical inhibition with a red-shifted microbial rhodopsin. *Nat. Neurosci.* **17**, 1123–1129 (2014). 24997763 10.1038/nn.3752 PMC4184214

[171] Han, X. et al. A high-light sensitivity optical neural silencer: development and application to optogenetic control of non-human primate cortex. *Front. Syst. Neurosci*. **5**, 18 (2011). 10.3389/fnsys.2011.00018 PMC3082132 21811444

[172] Ferenczi, E. A. et al. Optogenetic approaches addressing extracellular modulation of neural excitability. *Sci. Rep.* **6**, 23947 (2016). 27045897 10.1038/srep23947 PMC4820717

[173] Govorunova, E. G., Sineshchekov, O. A., Janz, R., Liu, X. & Spudich, J. L. Natural light-gated anion channels: a family of microbial rhodopsins for advanced optogenetics. *Science* **349**, 647–650 (2015). 26113638 10.1126/science.aaa7484 PMC4764398

[174] Hososhima, S. et al. A light-gated cation channel with high reactivity to weak light. *Sci. Rep.* **13**, 7625 (2023). 37165048 10.1038/s41598-023-34687-7 PMC10172181

[175] Mahn, M. et al. High-efficiency optogenetic silencing with soma-targeted anion-conducting channelrhodopsins. *Nat. Commun.* **9**, 4125 (2018). 30297821 10.1038/s41467-018-06511-8 PMC6175909

[176] Berndt, A., Lee, S. Y., Ramakrishnan, C. & Deisseroth, K. Structure-guided transformation of channelrhodopsin into a light-activated chloride channel. *Science* **344**, 420–424 (2014). 24763591 10.1126/science.1252367 PMC4096039

[177] Inoue, M. et al. Rational engineering of xcamps, a multicolor geci suite for in vivo imaging of complex brain circuit dynamics. *Cell* **177**, 1346–1360.e24 (2019). 31080068 10.1016/j.cell.2019.04.007

[178] Matlashov, M. E., Vera, J., Kasatkina, L. A., Khodakhah, K. & Verkhusha, V. V. Design and initial characterization of a small near-infrared fluorescent calcium indicator. *Front. Cell Dev. Biol.* **10**, 880107 (2022). 35846350 10.3389/fcell.2022.880107 PMC9277108

[179] Yokoyama, T. et al. A multicolor suite for deciphering population coding of calcium and camp in vivo. *Nat. Methods* **21**, 897–907 (2024). 38514778 10.1038/s41592-024-02222-9 PMC11093745

[180] Dana, H. et al. Sensitive red protein calcium indicators for imaging neural activity. *eLife* **5**, e12727 (2016). 27011354 10.7554/eLife.12727 PMC4846379

[181] Hao, Y. A. et al. A fast and responsive voltage indicator with enhanced sensitivity for unitary synaptic events. *Neuron* **112**, 3680–3696.e8 (2024). 39305894 10.1016/j.neuron.2024.08.019 PMC11581914

[182] Evans, S. W. et al. A positively tuned voltage indicator for extended electrical recordings in the brain. *Nat. Methods* **20**, 1104–1113 (2023). 37429962 10.1038/s41592-023-01913-z PMC10627146

[183] Lu, X. et al. Widefield imaging of rapid pan-cortical voltage dynamics with an indicator evolved for one-photon microscopy. *Nat. Commun.* **14**, 6423 (2023). 37828037 10.1038/s41467-023-41975-3 PMC10570354

[184] Platisa, J. et al. High-speed low-light in vivo two-photon voltage imaging of large neuronal populations. *Nat. Methods* **20**, 1095–1103 (2023). 36973547 10.1038/s41592-023-01820-3 PMC10894646

[185] Kralj, J. M., Douglass, A. D., Hochbaum, D. R., Maclaurin, D. & Cohen, A. E. Optical recording of action potentials in mammalian neurons using a microbial rhodopsin. *Nat. Methods* **9**, 90–95 (2012). 10.1038/nmeth.1782 PMC3248630 22120467

[186] Tian, H. et al. Video-based pooled screening yields improved far-red genetically encoded voltage indicators. *Nat. Methods* **20**, 1082–1094 (2023). 36624211 10.1038/s41592-022-01743-5 PMC10329731

[187] Kannan, M. et al. Fast, in vivo voltage imaging using a red fluorescent indicator. *Nat. Methods* **15**, 1108–1116 (2018). 30420685 10.1038/s41592-018-0188-7 PMC6516062

[188] Abdelfattah, A. S. et al. A general approach to engineer positive-going efret voltage indicators. *Nat. Commun.* **11**, 3444 (2020). 32651384 10.1038/s41467-020-17322-1 PMC7351947

[189] Song, C. et al. Characterization of two near-infrared genetically encoded voltage indicators. *Neurophoton*. **11**, 024201 (2023). 10.1117/1.NPh.11.2.024201 PMC10712888 38090225

[190] Geng, G. et al. Viral and non-viral vectors in gene therapy: current state and clinical perspectives. *eBioMedicine* **118**, 105834 (2025). 40602323 10.1016/j.ebiom.2025.105834 PMC12271757

[191] Wang, J.-H., Gessler, D. J., Zhan, W., Gallagher, T. L. & Gao, G. Adeno-associated virus as a delivery vector for gene therapy of human diseases. *Signal Transduct. Target. Ther.* **9**, 78 (2024). 38565561 10.1038/s41392-024-01780-w PMC10987683

[192] Eghbalsaied, S. & Kues, W. A. CRISPR/cas9-mediated targeted knock-in of large constructs using nocodazole and RNase HII. *Sci. Rep.* **13**, 2690 (2023). 36792645 10.1038/s41598-023-29789-1 PMC9931768

[193] Joo, J. H., Lee, S. & Kim, K. P. Precision gene editing: the power of crispr-cas in modern genetics. *Mol. Ther. Nucleic Acids* **36**, 102733 (2025). 41210586 10.1016/j.omtn.2025.102733 PMC12590234

[194] Gore, P. S. et al. Red-shifted excitation enhances the sensitivity of red genetically encoded ca2+ indicator and enables crosstalk-free two-photon holographic optophysiology. *J. Biomed. Opt*. **30**, (2025). 10.1117/1.JBO.30.11.116003 PMC12640144 41281773

